# Biotechnological Potential of Extremophiles: Environmental Solutions, Challenges, and Advancements

**DOI:** 10.3390/biology14070847

**Published:** 2025-07-11

**Authors:** Fabrizia Sepe, Ezia Costanzo, Elena Ionata, Loredana Marcolongo

**Affiliations:** 1 Research Institute on Terrestrial Ecosystems (IRET), National Research Council of Italy (CNR), Via Pietro Castellino 111, 80131 Naples, Italy; fabriziasepe@cnr.it (F.S.); ezia.costanzo@iret.cnr.it (E.C.); 2 Department of Veterinary Medicine and Animal Production, University of Naples, Federico II, Via Federico Delpino 1, 80137 Naples, Italy

**Keywords:** extremophiles, extreme conditions, biotechnology, biomedicine, sustainable development

## Abstract

Extremophiles are microorganisms capable of living on Earth in ecological niches characterized by peculiar conditions, including extreme temperatures and/or pH, high salt concentrations, and the presence of heavy metals. The development of unique structural and functional adaptation strategies has stimulated an increasing scientific interest since their discovery. The importance of extremophiles lies in their exploitability in significant bioprocesses with several biotechnological applications and their role as a fundamental source of numerous high-value-added biomolecules. This review aims to examine the diversity and specificities of extremophilic archaea and bacteria, with particular emphasis on their potential applications and development in biotechnology and biomedicine. The use of extremophiles and their extremozymes has allowed applications in several fields, such as bioremediation, sustainable agriculture, the recovery of bioactive molecules for use in bioenergy, biomedicine, and nanoparticle production. The comprehension and exploitation of the complex molecular mechanisms that enable life in extreme environments represent a challenge to mitigate current climate change problems and to invest in sustainable development towards a green transition.

## Simple Summary

The combined effects of dwindling global resources and an ever-increasing human population have led to a growing focus on sustainability issues. From this perspective, extremophiles, microorganisms that have evolved unique survival strategies for some of the most extreme environments on Earth, can be used in many areas of fundamental and applied science. This review explores the different types of extremophiles and their potential applications in biotechnology, including pollution remediation, improving agriculture, and producing materials useful for medicine and energy. Understanding how these microorganisms survive in such extreme conditions could help scientists to combat climate change and support a more sustainable, environmentally friendly future. Advanced technologies enable the development of innovative solutions that benefit both humanity and the planet.

## 1. Introduction

The term “extremophiles”, first coined by MacElroy in 1974 [1], refers to microorganisms that have developed the ability to grow and survive in ecological niches characterized by extreme and hostile conditions. In volcanic and polar regions, in acid and alkaline environments, in hydrothermal zones or underground mines, in oil fields, in radiation-contaminated sites, and even in the stratosphere, extremophiles can be found everywhere on Earth. In every extreme environment studied, wide ranges of organisms have shown that they can not only tolerate extreme physico-chemical conditions but also require them for survival.

In the mid-1960s, Brock and his collaborators isolated *Thermus aquaticus* [2], a filamentous bacterium living at high temperatures (75 °C) in Yellowstone National Park, sparking a growing interest in extremophilic organisms. This offered a unique opportunity to explore the evolutionary origins and limits of life, helping to define the environmental thresholds by which it is supported.

**Figure 1:**
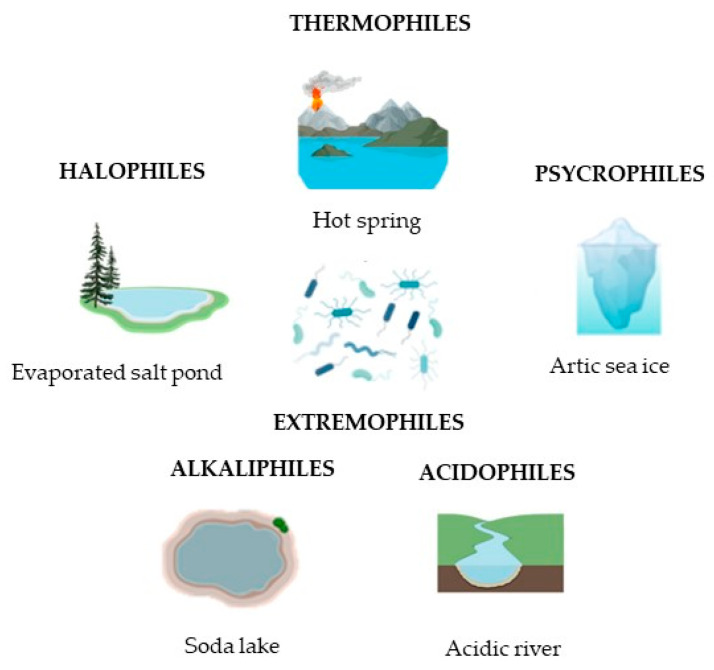
Different habitats of extremophiles. Image created with BioRender.com.

Extremophiles are classified according to their different peculiarities (Figure 1). Thermophiles and hyperthermophiles live at high temperatures, while psychrophiles tolerate low temperatures; acidophiles/alkalophiles inhabit very acidic or alkaline environments; halophilic microorganisms tolerate environments with high salt concentrations, or piezophiles, which are able to grow at the high pressures typical in ocean depths; metallophiles, which thrive in the presence of metal and heavy metals; oligotrophic microorganisms, which are capable of growth in nutrient-poor habitats; xerophiles, which are resistant to desiccation [3]. Furthermore, such microorganisms are often tolerant to more than one extreme condition and are categorized as poly-extremophiles, which are found in various extreme ecological niches [4].

The scientific attraction for extremophiles was also due to their role as an important reservoir of genetic diversity, unique metabolic pathways, and biological molecules that enable them to cope with multiple environmental stresses. Moreover, enzymes produced by extremophiles, known as extremozymes, are able to work efficiently under the environmental conditions in which extremophilic microorganisms live [5]. This has led to research into these new extremophilic biocatalysts, which exhibit stability as well as high efficiency and robustness under the denaturing conditions for mesophilic enzymes and can be used in a wide range of harsh biotechnological contexts. One of the most important and successful examples of extremophilic products is enzymes for DNA manipulation (Taq polymerase) [6], but there are numerous extremophilic enzymes that are crucial in large-scale biotransformation, such as drug biosynthesis, cosmetic formulations, or detergents. Indeed, the global direction of research is aimed at the implementation and optimization of sustainable biotechnological, agricultural, and industrial processes, which are methodologies for the monitoring and elimination of environmental pollutants and mitigation of global warming, under the impetus of the 2030 Agenda for Sustainable Development (United Nations) [7]. In this context, there is a need for in-depth research into the properties and potential of extremophilic microorganisms, a necessary requirement for the development of white and green biotechnologies in the industrial, biomedical, and bioenergy fields, the prevention and remediation of environmental pollution, and the production of biomaterials, moving towards a bio-based economy [5].

Although extremophiles are already being used in many areas of biotechnology and, as will be discussed below, have enormous potential for current and future applications, it needs to be added that the exploitation of these organisms is not without bottlenecks.

The harsh conditions under which some extremophiles grow, as well as the costs involved in producing and scaling them up, drive the need to develop efficient solutions, including genetic engineering of microorganisms with characteristics desired for biotechnological applications [8]. There are countless examples of extremophilic microorganisms, mostly prokaryotic (archaea and bacteria), with less representation from eukaryotic organisms. In this regard, the focus of this review is on the archaeal and bacterial sources of extremophiles and the past and present status of their ecological significance. It also aims to highlight some significant applications in the many potential fields of microbial biotechnology.

## 2. Extremophiles

The main groups of extremophiles listed in this section have been reported in Table 1.

### 2.1. Thermophiles

Microorganism adaptation to temperature has always been one of the most intriguing areas of research in determining the limits of life on our planet [51]. The lowest and highest temperatures on Earth are −98 °C and 495 °C, respectively, measured in locations in East Antarctica and deep-sea hydrothermal vents. However, life at extreme temperatures is only possible in the presence of liquid water, so life forms that can develop at temperatures higher than 100 °C must also be resistant to high pressure. Actually, despite the theoretical limits for life spans ranging from −40 and 150 °C, defined on the basis of thermodynamics and macromolecules’ stabilities, the real thresholds at which microorganisms survive and show metabolic activity are −20 and 122 °C. In particular, at 122 °C, the growth of *M. kandleri* was demonstrated, while at −20 °C, the metabolic activities of the cryptoendolithic bacteria were measured in Antarctic permafrost [52].

Temperature gradients greatly determine microbial distribution, especially in thermophilic environments, where the effects of increasing temperatures on microbial adaptations are particularly studied. With respect to the limits of temperatures tolerated by microorganisms, they are grouped in moderate thermophiles (thriving above 45 °C), extreme thermophiles (between 65 and 80 °C), and hyperthermophiles (above 80 °C) [53]. Most of the thermophilic and hyperthermophilic microorganisms belong to the archaea domain, but several other forms of bacteria have also been studied.

Different species of archaea have been identified in environments where temperatures exceed 100 °C due to geothermal activity and high pressure. The main environments inhabited by hyperthermophiles are terrestrial hot springs, deep-sea hydrothermal vents, and solfataric fields in shallow waters. In these ecosystems, microorganisms have developed an anaerobic metabolism due to low oxygen levels and the presence of inorganic-oxidizing or -reducing compounds. Moreover, they have adopted life strategies such as chemoautotrophy or chemolithoheterotrophy that obtain energy from chemical reactions on inorganic substrates but utilize different carbon sources represented by CO_2_ or organic compounds, respectively. Thermophilic organisms can be found in compost heaps, sunny soils, and man-made environments such as laundries, water heaters, and industrial plants [54]. Archaea predominate in warmer habitats such as deep-sea hydrothermal vents, where there are large temperature differences ranging from 400 °C near the vents to 2–4 °C in the surrounding seawater. Methanogens euryarchaeota, such as *M. jannaschii* and *M. kandleri*, are able to withstand the high temperatures and pressures found in deep-sea volcanic environments. *M. jannashii*, isolated from a sample collected near a “white smoker” chimney on the East Pacific Rise at a depth of 2600 m, was the first studied hyperthermophile from submarine hydrothermal environments [9]. *M. kandlerii*, isolated from hot deep-sea sediments [10], together with *M. jannashii*, show an anaerobic and chemoautotrophic metabolism, utilizing CO_2_ and H_2_ as carbon and energy sources, and are able to withstand 122 and 85 °C, respectively, and pressures of up to 20 MPa [11]. Moreover, chemolithoautotrophic *G. barossii*, a Fe III reducer [12], is the second most thermophilic microorganism after *M. kandlerii* since it lives at 121 °C, and is, therefore, named strain 121. Several species of hyperthermophiles belonging to the *Pyrococcus* genus, such as *P. furiosus*, *P. horikoshi*, and *P. abyssii*, living in hot marine environments, are also being studied. These sulfur-metabolizing, chemolithoheterotrophic archaea survive and reproduce at temperatures around 100 °C at an elevated pressure (15 and 20 MPa for *P. horikoshi* and *P. abyssii*), with the sole exception of *P. furiosus*, which exhibits an optimal temperature for growth at 100 °C and 0.1 MPa [13,14,15].

Terrestrial geothermal sites (hot springs and solfataric fields) also harbor thermophic bacteria and archaea. *Acidianus* and *Sulfolobus* are crenarcheota genera widely represented in these habitats characterized by high temperatures and low pH. Following the isolation of the first *S. acidocaldarius* from a hot spring in Yellowstone National Park [2], other species were discovered, such as *S. solfataricus* from the Pisciarelli solfatara fields in Naples [17] and *S. islandicus* [17] and *S. tokodaii* from hot springs in Japan [18]. The common features are their thermo-acidophilic nature that allows them to grow within pH and temperature intervals of 0.9–5.8 and 55–80 °C and a predominant aerobic chemoorganotrophic metabolism on sugar and proteins. They are also chemoautotroph microorganisms that utilize, as an energy source, the oxidation of sulfur to sulfuric acid, which accumulates in their environments [55]. Among the bacteria most represented are those belonging to the *Aquificaceae* and *Thermotogaceae* families. Bacteria belonging to the *Aquifex* genus are among the most thermophilic bacteria (95 °C for *A. eolicus* and *A. pyrophilus*) that were isolated from hot marine sediments [16]. The *Thermotoga* genus comprises nine Gram-negative species of anaerobic bacteria that are extremely thermophilic, isolated from geothermally heated marine and terrestrial environments [56]. Their growth temperatures range from 65 to 80 °C, with an optimal pH around neutral. Their metabolism is chemolithoheterotrophic and prefers sugars fermented with the production of H_2_.

Huber et al. [19] isolated the type strain *T. maritima* from the volcanic areas of the Vulcano and Ischia islands and Naples Bay. This microorganism strain has an optimal growth temperature of 80 °C but can survive up to 90 °C and belongs to Eubacteria; these elevated temperatures are typical of archaea.

Another species that shows elevated structural similarities, e.g., a membrane lipid composition and the same optimal physico-chemical parameters for growth, is *T. neapolitana*, which was isolated independently in the same years and areas (Lucrino bay near Naples) and was recognized as a different species by DNA hybridization [20]. Other remarkable thermophilic bacteria species (60–80 °C) are distributed among numerous genera, such as *Clostridium*, *Bacillus*, *Paenibacillus*, *Alicyclobacillus*, *Anoxybacillus*, *Thermus*, *Rhodothermus*, *Thermobifida*, *Geobacillus*, that occupy various ecological niches in different aquatic, terrestrial, and anthropized environments [57]. The enormous microbial diversity in (hyper)thermophilic environments, which was only briefly discussed above, requires complex and challenging biochemical and physiological adaptations. Cell membrane integrity and fluidity are crucial issues at increased temperatures and can be guaranteed by enhancing the transition temperature of the lipid fraction through modulating the fatty acid composition. In bacterial membranes, saturated fatty acids with higher lengths and ramifications are preferred, as are those that have terminal cyclo-groups (cyclohexyl groups), which restrict mobility in the middle of the bilayer. The particular composition of membranes of (hyper)thermophilic archaea involves, instead, the substitution of fatty acids with isoprenoid chains that are ether-linked with two glycerol molecules (tetraether lipids) that span the entire membrane thickness originating from a single-layer structure [58]. These dialkyl diglycerol tetraethers enable industrial enzyme stability in organic solvents.

The molecular adaptations that ensure resistance and rigidity are also functional to the critical issue of regulating the membrane permeability via maintenance at constant levels of the proton permeability, which becomes faster when the temperature increases [59]. At the genomic level, the most widely used strategies involve reducing the size of thermophilic genomes compared to mesophilic ones by shortening non-coding and coding sequences and eliminating entire genes, allowing for easier genome replication.

Moreover, the increased frequencies of G and C bases, as well as the presence of more efficient DNA repair strategies, lead to greater nucleic acid stability and integrity. The modified frequencies of certain codons are evident with the preference towards NTN types that encode hydrophobic amino acids, such as valine, isoleucine, leucine, and methionine [60]. The unusual abundance of A- and G-rich terminal sequences leads to the predominant synthesis of charged amino acids, which, together with hydrophobic amino acids, improve protein thermostability. Furthermore, the predominant features ensuring protein resistance to high temperatures are the abundance of salt bridges and disulfide bonds, along with the presence of extended hydrophobic interactions and charged surfaces [61].

### 2.2. Psicrophiles

Psychrophyles are microorganisms that live in cold environments below 20 °C, with an optimal growth temperature below 15 °C. They are classified into two subcategories: psychrotrophs or psychrotolerant, which have maximum growth rates above 15 °C and can survive below 0 °C; psychrophiles, which prefer temperatures below 15 °C and cannot grow above 20 °C [62]. The actual lower limit for metabolically active psychrophiles is −20 °C, while pure cultures were able to grow at −12 and −15 °C, as reported for *P. ingrahamii* and *P. halocryophilus* Or1, respectively [21,22]. Due to their wide growth temperature ranges (−20 °C to 20 °C), psychrophiles represent the most widespread extremophilic microorganisms, which are distributed over 75% of our planet (aquatic and terrestrial ecosystems) that is permanently cold with temperatures under 5 °C [62]. A clear idea of the high extension of cold environments is just from the only consideration that oceans cover 70% of Earth’s surface, with 90% of water mass at 5 °C or below due to constant temperatures of 4–5 °C reached under the thermocline regardless of latitudes. The fundamental ecological role of psychrophiles, which are responsible for biogeochemical cycles in the cold biosphere, is reflected by their presence in Polar regions (Antarctic subglacial lakes and glaciers, Arctic permafrost, and ice sheets), deep-sea areas, high-altitude mountains, and drops of water in upper-atmosphere clouds [23]. The multiple harsh and challenging conditions to which the cold-adapted microorganisms are exposed have often prompted the development of poly-extremophilic characters. In deep-sea environments, piezophilic psychrophiles have evolved to withstand elevated pressures. Different piezo-psychrophilic species belonging to the Proteobacteria genus are reported, such as *S. benthica* [63] and *C. hadaliensis* [24], which were the first obligate piezophiles isolated from deep-sea environments at 7500 and 10,000 m depth and require growth pressures of 50 and 70 MPa, respectively.

Several cold-adapted species belong to the genera *Photobacterium*, *Moritella*, and *Psychromonas*, including *M. yayanosii* [25], which has been reported as one of the most piezophilic bacteria isolated in the Mariana Trench Challenger Deep at a depth of 10,898 m and is capable of withstanding pressures up to 100 MPa [24]. Poly-adaptation strategies are also those evolved by halo-psychrophiles, which are well-represented microbial consortia inhabiting hypersaline veins or liquid films in glacial ice.

In these brine pockets, especially those surrounding soil particles rich in inorganic and organic nutrients, sub-zero temperatures of up to −40 °C are reached, along with high salt concentrations, following salt exclusion from ice crystals [64]. Microbial cells are subjected to internal solute concentrations by osmotic mechanisms and undergo vitrification status, avoiding intracellular freezing and allowing microorganism survival and metabolic activities up to −20 °C [65]. The considerable bacterial biodiversity in cold ecosystems is the result of numerous strategies adopted by psychrophiles to cope with several stressful conditions, among which intracellular freezing is one of the most challenging. The production of intracellular anti-freezing proteins (AFPs) (glyco and lipoproteins) is the effective adaptive mechanism exploited by several Antarctic species, such as *M. cryophilus*, *R. erythropolis*, *M. protea*, *M. primoryensis*, and different *Moritella* spp. [26,27]. AFP proteins reduce the liquefaction temperature of intracellular fluids, thus preventing the nucleation of large crystals or their formation by inhibiting ice recrystallization at sub-zero temperatures. Studies by Rahman et al. [66] also demonstrated the production of extracellular AFPs by psychrophilic *Sporosarcina* and *Polaromonas* spp., which lower the freezing point of saline liquid films outside the cells and stabilize the brine pocket environments. Moreover, AFP forms a protective outer layer for the outer membrane and entire cell, interacting with capsular and membranous polysaccharide structures [67]. An effective cryoprotection role is demonstrated by exopolysaccharides (EPS) and compatible solutes such as threalose, glycerol, mannitol, glycine, and betaine. In particular, threalose is capable of counteracting protein aggregation, scavenging free radicals, and preserving the cell membrane structure [68]. Furthermore, EPS production is reported to promote cell adhesion and biofilm formation, which lowers the freezing point around the bacterial cells [69]. In this mechanism, the quorum-sensing of cell-to-cell communication plays a crucial role by regulating the expression of several genes, among which are those for biofilm formation, cell adhesion, and motility [70]. Other remarkable microbial survival mechanisms involve adaptations of the cell membrane to low temperatures with increased fluidity and permeability of the outer envelopes. The prevalence of polyunsaturated fatty acids with double bonds, preferentially in cis isomeric form, leads to short-chain fatty acids and a reduction in the charge and dimensions of phospholipid heads, which effectively prevents the negative effects of membrane fluidity from exposure to cold temperatures. In addition, the hyper-expression of genes coding for membrane proteins maximizes the efficiency of transportation within the bacterial cells of nutrients, while those for the production of compatible solutes allow for counteracting the low diffusion rate and external effects of hyper-osmolarity [70]. In cold ecosystems, the presence of carotenoids also modulates membrane fluidity adjustments in response to temperature fluctuations. Due to the importance of temperature on the rates of enzymatically catalyzed metabolic reactions, the specific adaptation of enzymes is directed to minimize the effects of low temperatures [71]. An enhanced molecular flexibility is ensured by weakening intramolecular and inter-subunit interactions via a reduction in hydrogen bonds, salt bridges, and hydrophobic interactions. In addition, as the length of the cycle structures increases, hydrophobic interactions with the solvent lead to more flexible structures and greater accessibility of the active site, which is functional for achieving highly specific activities at low temperatures [72]. A protein composition that favors amino acids with small, neutral side-chains, such as asparagine, methionine, and glycine, also favors a less ordered and more flexible structure [71]. Additionally, the protein expression profile of psychrophilic microorganisms is modified through the regulation of genes that code for antioxidant enzymes (e.g., catalase and peroxidase), which protect cells from damage caused by reactive oxygen species (ROS) produced by increased oxygen solubility. Cold-shock proteins are also hyper-expressed. These single-stranded nucleic-acid-binding molecules enhance transcription and translation rates, aiding the destabilization of secondary DNA and RNA structures [72].

### 2.3. Acidophiles

Acidophiles are microorganisms that thrive in natural and anthropized environments characterized by low pHs, such as acid volcanic areas (solfataric fields and sulfuric pools), marine hydrothermal vents, acid mines drainages, acid rock drainages, and animal stomachs [73]. The ecosystems located in volcanic or geothermal areas such as Yellowstone Park (USA), the Solfatara supervolcano (Italy), and the hot springs on the Azores islands, among others, are characterized by the presence of abundant sulfurous compounds emissions. Acidophiles have strict adaptation requirements as they can only grow within a narrow pH range of two units above their optimal value and cannot tolerate a neutral pH. They are classified into moderate acidophiles, which show optimal pHs for growth between 5.0 and 3.0, and extreme acidophiles, which are adapted at pH values of 3.0 or below [28]. Acidophiles are abundant in environments such as mine and rock drainages that are rich in sulfide and iron minerals, which they oxidize, and the biogeochemical activity contributes to the acidic pH of the ecosystems. *Acidithiobacillus* genus harbors chemoautolithotrophic acido-tolerant species such as *A. thiooxidans* [28] and *A. caldus* [29], which are able to oxidize sulfur compounds, while *A. ferroxidans* is has been studied as a psychrotolerant sulfur/iron-oxidizing species that dominates pyrite mines [30]. Moreover, *A. ferrooxidans* and *L. ferrooxidans* [31] can also reduce iron, as demonstrated by strains of these bacteria isolated from acidic lakes and mine runoffs, as well as from the Spanish river Rio Tinto, whose waters reach a pH of 2.0 in the presence of heavy metals [74].

Consequently, the microorganisms that colonized the area evolved particular resistance mechanisms to cope with these elevated concentrations of toxic metals and developed the ability to dissolve and extract ferrous minerals through oxidation, thus attracting huge biotechnological interest in biomining applications [75]. The combination of a low pH and elevated temperatures is prevalent in hydrothermal ecosystems such as solfataric fields and sulfuric pools that are ideal habitats for thermos-acidophilic species. The most acidophiles reported so far are the heterothophic archaeal species *P. torridus* and *P. oshimae* (optimal pH ≤ 0.7) that grow optimally at 60 °C [32], while hyperthermophiles (T 80 °C, pH 3.0) belong to the *Sulfolobales* order of *Crearchaeota* [2]. Among these species are *S. solfataricus* and *S. acidocaldarius*, which can be easily grown in the laboratory and have been widely studied at the genomic, biochemical, and metabolic levels. In contrast, *A. brierleyi*, *A. infernii*, and *M. sedula* were investigated for their potential in bioleaching [34]. The capacity of extreme acidophiles to withstand a low pH is even more surprising when considering their evolved protective mechanism of maintaining the internal pH around neutrality, where a reduced proton permeability of the cell membrane is an effective strategy adopted by both bacteria and archaea. The change in the composition of the phospholipid bilayer in acidophilic bacteria, which is achieved by favoring a higher concentration of branched unsaturated fatty acids with terminal cyclohexyl and cyclopropane groups, improves the membrane compactness and limits proton permeability [76]. The archaeal cell membrane, consisting of a monolayer of tetraether lipids, acts as a highly effective barrier to a proton influx. The lower sensitivity of ether linkages to acid hydrolysis compared to ester bonds improves the membrane’s resistance [73]. The reduction in the size of membrane protein channels for proton entry and the improvement in H^+^ efflux system pumps (H^+^ ATPases, symporters, and antiporters) are also widely demonstrated mechanisms by which acidophiles avoid cytoplasmic acidification [77]. Moreover, the proton entrance is inhibited by the inversion of trans-membrane potential, which becomes positive inside by exploiting a higher K^+^ influx with respect to an H^+^ efflux. This is demonstrated by a significant presence of genes coding for K^+^ and other cation pump proteins in genomes of acidophiles, such as *T. thiooxidans* and *P. torridus* [33,35]. The cytoplasm buffering capacity is also enhanced to counteract an intracellular excess of protons. This is achieved by increasing the presence of basic amino acids (e.g., lysine, arginine, and histidine) and the expression of enzymes (decarboxylase) that require protons to be active [78]. The need for repair systems for DNA and proteins damaged by acidity is of vital importance for cell survival. An elevated number of genes coding for enzymes involved in DNA and protein repair, as well as chaperones for protein refolding, has been widely reported for several species [79]. Effective quorum-sensing systems that allow the proper regulation of biofilm composition and formation are crucial for the growth of microorganisms in these challenging environments [80].

### 2.4. Alkaliphiles

Alkaliphiles are extremophilic microorganisms that live in alkaline media at pH ≥9.0 and show limited or no growth near neutrality (pH 6.5) [3]. Among them, obligate alkaliphiles require at least pH 9.0 and show the highest growth rates at pH 10.0, while for facultative alkaliphiles, pH <8.0 is tolerated, but they show maximal growth at pH 10.0. Alkali-tolerant microorganisms grow optimally at pH 7.0 and can also flourish at pH 9.0, but cannot survive at pH 10.0–11.0 [81].

Different from the other extremophiles, microorganisms adapted to alkaline pH can share the same habitats with neutrophilic species. Neutral soil communities harbor 1–10% of alkaliphilic microorganisms, mainly belonging to *Bacillus* species that thrive in microenvironments at a pH of around 10.0, generated by a sulfate reduction and ammonification of the urea. The elevated number of facultative alkaliphiles determines the presence of these species in ecosystems where microhabitats characterized by transiently alkaline conditions are scattered in neutral soil areas [3]. Elevated pH levels are also caused by industrial activities like cement manufacturing (high Ca(OH)_2_ concentrations), mining, paper and pulp production (high NaOH concentrations), indigo fermentation, electroplating, and hide and food processing [82]. High pH levels of around 9.5, transiently generated by natural silicate decomposition, are also found in alkaline hot springs [83]. Stable alkaline environments are characterized by the greatest diversity of alkaliphiles. Alkaline soda lakes, with high concentrations of calcium ions (Ca^2+^), are mainly located in arid tropical regions, such as the East African Rift Valley and Central Asia, but they are also present in America, Europe, and Australia. In these sites, surface water evaporation determines high concentrations of sodium carbonate (pH 8.5–12.0) and elevated NaCl concentrations (5–30%), producing halo-alkaline environments. Conversely, Ca^2+^-rich groundwater, found in a few locations in Turkey, Oman, California, Jordan, and Cyprus, is caused by the decomposition of calcium and magnesium silicates, which release CaOH and cause pH levels to rise to around 11.00 [84]. Studies of the diversity of alkaliphilic bacteria began with the isolation of the first species, *S. pasteurii* (formerly *B. pasteurii*), followed by *B. alkaliphilus* [38]. Several other *Bacillus* spp. were subsequently isolated and identified until the end of the 1990s, when the discovery of biodiversity in African soda lakes opened up much wider studies of alkaliphile species and their classification [81]. Soda lakes are the most studied alkaline environments, and their biodiversity includes species from most alkaliphilic and haloalkaliphilic bacterial and archaeal groups. Several bacterial spp. belonging to the phyla *Firmicutes* and *Proteobacteria* populate these ecosystems, mainly belonging to the genera *Bacillus*, *Paenibacillus*, *Alkalibacillus*, *Pseudomonas*, *Alkalymonas*, *Halomonas*, and *Marinobacter*, among others. Species such as *B. alkaliphilum* [36], *B. halodurans* [42], *B. natronophilus* [37], *N. bacteriovora* [38], *M. alkaliphilum* [43], and *N. tapanii* [44] are among the most-studied species. Photosynthetic cyanobacteria are the most prevalent species in soda lake habitats, where they are primary producers with productivity rates of up to 10 g/cm^2^/day. The intense blue-green bloom of cyanobacteria, such as *A. fusiformis* and other *Arthrospira* spp., is caused by high light intensities, temperatures, and unlimited CO_2_ availability. This leads to cell counts of 13,000 bacterial filaments/mL, placing soda lakes among the aquatic ecosystems with the highest biomass yields [45]. Anaerobic, sulfur-reducing halo-alkalophilic purple bacteria that use H_2_S as an electron donor instead of H_2_O for their anoxygenic photosynthesis also contribute to primary production and N_2_ fixation in soda lake ecosystems. The thermophilic halo-alkaliphilic *H. halochloris* and *H. halophila*, belonging to the *Chromatiales* order, are among the purple photosynthetic bacteria isolated from soda lakes [40,46]. In sulfuric biogeochemical cycles in soda lakes, anaerobic sulfate-reducing bacteria (SRB) utilize sulfate, thiosulfate, and sulfur, which serve as electron acceptors that are responsible for sulfidogenesis with H_2_S production [85]. They thrive in the non-oxygenic environment of soda lake sediments with species belonging to the orders of *Desulfovibrionales* and *Halanaerobiales*, such as *D. hydrogenovorans* [39], *D. lacustre* [41], *D. thiodismutans* [38], *D. zhilinae* [47], and *H. betaine* [48] among others. Sorokin et al. [86] also discovered methanogenesis as an anaerobic respiration mode adopted in the sediment zone of meromictic soda lakes. The alkaliphilic archaeal genera *Methanolobus*, *Methanosalsum*, and *Methanocalculus* have been studied in relation to methanogenic metabolic pathways utilizing methylated carbon compounds or hydrogen as substrates and electron donors [87]. Among archaeal species belonging to *Natronobacterium*, *Natronomonas*, *Natrialba*, and *Natronococcus* genera [88], aerobic haloalkaliphiles characterized by pink-pigmented cells, with densities of up to 10^7^–10^8^ cells/mL, can be reached in soda lake brines, resulting in pink-red blooms.

The plentiful life with an alkaline pH prompted intensive study efforts to unravel the solutions at cellular and molecular levels adopted by microorganisms to cope with these challenging environmental conditions. Alkaliphilic bacteria stabilize the intracellular pH to about two units below that of the environment. This is achieved by modifying cell envelopes to retain H^+^ near the cell surface, lowering pH and enhancing the barrier effect [44]. A strategy to strengthen cell walls and membranes includes increasing negatively charged compounds like teichuronic acid, peptides, and poly-γ-D-glutamic acid, and lowering unsaturated fatty acids. High squalene and anionic phospholipid levels strengthen cell membranes against H^+^ leakage [89]. Alkaliphiles survive by producing acids in the cytoplasm through the expression of enzymes such as amino acid deaminase and oxidoreductase. Organic acids are also produced through sugar fermentation and acidic amino acids. To counteract the rising cell internal pH, membrane transporter proteins are expressed, such as monovalent cation/proton antiporters and ATP synthases [44].

### 2.5. Halophiles

Hypersaline environments, such as salt lakes, evaporation ponds, polar ice brine channels, salty foods, saline soils, and marine environments, are widespread. The Dead Sea, the Great Salt Lake, and the brine pools of the Red Sea and the eastern Mediterranean are the best-characterized [90]. Halophilic microorganisms that inhabit hypersaline environments have developed life strategies based on the presence of salt, which is required for their growth and cellular integrity. Based on salinity levels, they can be categorized as extreme or moderate halophiles, which grow maximally in the presence of NaCl at concentrations of 15–30% and 3–15%, respectively, or as weak halophiles, which comprise marine species that require concentrations of 1–3%. Microorganisms that do not require salt are also capable of a high level of tolerance, with NaCl concentrations ranging from 1 to 15 [91]. Halophile distribution spans from archaea to bacterial species. The latter are included in mixed taxonomic groups that also comprise mesophilic members, with the exception of the *Halanaerobiales* order that contains only halophilic aerobic bacterial spp. Archaeal halophiles, also known as *Haloarchaea*, are mainly grouped in the homogeneous order of *Halobacteriales*. These aerobic chemoorganoeterotrophic microorganisms, which are included in *Halobacterium* and *Halococcus* genera, require elevated NaCl concentrations (20–25%) and undergo cell lysis at NaCl concentrations below 10–15% [91]. In addition, some halophilic anaerobic methanogenic archaeal spp. belonging to *Halomethanococcus*, *Methanohalophilus*, and *Methanohalobium* genera, which are classified in the mixed order of *Metanosarcinales*, have been described [92]. Haloarchaea are the prokaryotes best adapted to high concentrations of sodium chloride (NaCl); they predominate in hypersaline environments characterized by limited biodiversity and are mainly restricted to extremely halophilic archaea. These microorganisms often exhibit poly-extermophilic characters (acidophilicity, alkalophilicity, thermophilicity, and psychrophilicity) and have different morphologies (cocci, rods, pleomorphic, or unusual shapes such as triangles) [93] and cause fascinating red-pink colorations in habitats such as salt lakes and pans. The retinal membrane proteins (bacteriorhodopsin, halorhodopsin) that function as light-driven protons and chloride pumps, and bacterioruberin are typical red-pink C50 membrane carotenoids produced by *Halobateria* spp., with the sole exception of some *Natrialba* spp., and are responsible for these wonderful spectacles [93]. Halophilic bacteria show a remarkable plasticity in their adaptability to wide ranges of salinity levels that span from moderate to extreme conditions. They are included in several groups like *Firmicutes*, *Cyanobacteria*, *Proteobateria*, *Thermotogae*, *Actinobacteria*, and *Bacteroidetes* [94]. They coexist with archaeal species and become predominant as the salinity gradient decreases, adopting specific mechanisms to address the hyper-osmotic challenges of an external medium.

The main strategy used by bacteria to balance osmotic pressures and maintain low salt concentrations inside the cytoplasm is to use organic ‘compatible’ solutes. These molecules, which consist of sugars (e.g., trehalose and sucrose), amino acids and their derivatives (e.g., glycine betaine, glutamate, proline, and ectoine), and alcohols (e.g., glycosylglycerol), can be used in combination. They can be produced internally or imported from the medium, where specialized microbial community members secrete them [95]. Elevated energy amounts are required not only for the synthesis of organic molecules but also for the adequate functioning of Na/H^+^ antiporter systems that allow keeping NaCl outside the cell, thus avoiding cytoplasmic protein aggregation through salting out [91]. Bacteria such as *H. elongata*, *M. marina*, and *M. terricola* [49], as well as most cyanobacteria, are able to synthesize organic osmo-protectants. In contrast, *S. salinus* and *C. salexigens* import compatible solutes, such as glycine betaine, arsenobetaine, and ectoin, from the medium [50]. The second osmo-protective strategy, which is more energy-efficient, involves the accumulation of K^+^ inside the cytoplasm and the extrusion of Na^+^. This mechanism, which has required the gradual evolution of numerous complex adaptations to allow intracellular enzymatic proteins to function at high salt concentrations, is mainly exploited by archaeal microorganisms in the *Halobacteriales* order and anaerobic bacteria in the *Haloanaerobiales* order. In a K^+^-rich cytoplasmic environment, acidic proteomes are retrieved, as halophilic proteins are protected from destabilization by enhanced hydrophobic interactions with large negatively charged surfaces. The repulsive forces between charged particles and the hydrophilicity of the anionic carboxyl groups on the surface preserve protein flexibility and hydration, thereby favoring protein activity even at high salt concentrations [96].

## 3. Applications

**Figure 2:**
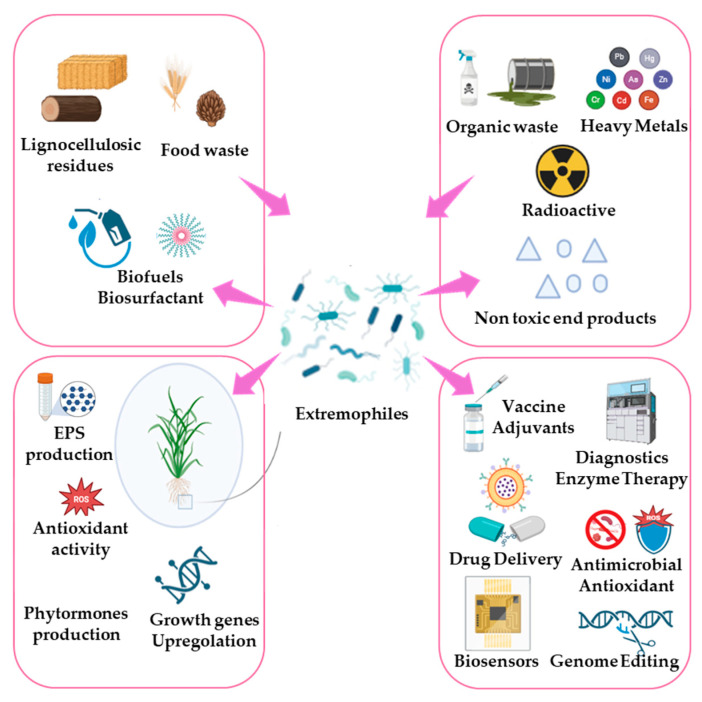
Application of extremophiles in biotechnology. Image created with BioRender.com.

The extremophiles described are able to thrive in extreme conditions and have developed remarkable survival mechanisms. These unique properties make these microorganisms particularly interesting for a wide range of biotechnological methodologies. Numerous applications (Figure 2) have already been discovered and below analyzed, but these intriguing microorganisms still offer possibilities to be fully explored.

### 3.1. Bioremediation

Bioremediation represents an attractive methodology to reduce pollution due to its sustainability, cost-effectiveness, and high efficiency. The key characteristics of extremophiles in the bioremediation process involve the microorganism’s adaptability to environments with high levels of pollutants. The enzymatic stability and activity under harsh conditions, such as high temperatures, extreme pH, and high salinity, allow them to degrade pollutants without undergoing denaturation. Furthermore, extremophiles possess unique metabolic pathways that enable them to transform a wide range of hazardous chemicals, including hydrocarbons, phenolic compounds, and heavy metals, into less toxic forms [75]. Environmental bioremediation processes require several catalytic steps and are most advantageous when performed using whole microorganisms. Over the years, numerous organisms such as archaea and bacteria, as well as yeasts and fungi, have been studied and used in degradation and/or transformation processes to mitigate the damage caused by environmental pollutants. Sites are often contaminated with different types of pollutants, and the use of extremophiles that cooperate in chemical transformation processes can be applied with great success [97].

#### 3.1.1. Heavy Metals

One of the areas of interest for the application of extremophilic microorganisms is the treatment of heavy metals (HMs). They are released from numerous urbanization and industrialization activities, in particular from mining, industrial, agro-industrial (fertilizers), and municipal waste (electronic device batteries, and paints), as well as vehicle emissions [98]. HMs are among the most toxic pollutants because they are non-biodegradable and persistent in the environment, and even at low concentrations, they are dangerous to the health of living beings and the environment [99]. Microbial processes of metal biosorption, bioaccumulation, bioaugmentation, and biomining are part of biometallurgy, which is the study of the interaction between microorganisms and metal ores or minerals. Combining bioremediation with metal recovery would have the dual benefit of reclaiming polluted sites and recovering important materials, such as rare-Earth metals [99]. Among the extremophilic microorganisms, acidophilus are able to recover HMs from industrial waste due to their ability to mobilize numerous elements, as in the case of the *Acidithiobacillus* species [100]. *Acidithiobacillus ferrooxidans*, a hyper-acidophilic extremophile, oxidizes iron and sulfur compounds through its specialized metabolism and easily solubilizes various trace elements. These characteristics were used in biomining and bioleaching processes since they are able to mobilize elements such as Lithium, Chromium, Nickel, and Copper, as well as Lead and Uranium [101]. *A. ferrooxidans* under ferrous iron oxidation was able to extract up to 100% of Zn, Co, Cu, and Mn from incineration residues [102]. *A. thiooxidans* has has also been shown to be effective in leaching metals, achieving a solubilization rate for Cr, Zn, Cu, Pb, and Cd in contaminated soils between 11 and 99%. Furthermore, about 89% of Ni was leached from spent oil catalysts. Interestingly, this acidophilic microorganism promoted the dissolution of plutonium in contaminated soils through continuous acid production [28].

The chemoautotrophic *A. thiooxidans*, *A. ferrooxidans*, and *L. ferrooxidans* were tested with heterotrophic bacteria that were Fe/S-oxidising (*Acidiphilium cryptum*) [31] for the bioremediation of sediments contaminated with HMs. The bacterial consortia enhanced the HMs removal, with an extraction yield of about 90% for Cu, Cd, Hg, and Zn. The direct reduction in vanadate and the recovery of vanadium were explored using *Acidocella aromatica* PFBC [103], while the acidophilic microorganism *Acidiphilium symbioticum* H8 bound Cd cations through an electrostatic reaction and complex formation [104]. Interestingly, the efficiency of chromium reduction by *Halomonas smyrnensis* KS802, a halophilic bacterium, was investigated [105]. The Cr(VI) of a tannery effluent was completely reduced in 6 h in the presence of a saline environment and other toxic metal ions. The consortium of metal-hyper-resistant microorganisms includes *Sulfobacillus thermotolerans*, which, in the presence of high zinc concentrations, shows an efficient zinc uptake together with low intracellular accumulation [106]. *Sulfobacillus thermosulfidooxidans* showed a remarkable adsorption capability of Cd, Cu, Ni, and Zn in the presence of acid mine drainage [107].

From both an economic and biosustainable perspective, the recovery of rare elements is of strategic importance. *Thermus scotoductus* SA-01 was able to accumulate Europium, survive, and grow in its presence (up to 1 mM). From this perspective, the thermophilic microorganism may have potential applications in the biorecovery of Eu and rare-Earth metals from geothermal niches [108]. In particular, Ozdemir et al. [109] observed the effects of the HMs Cd, Cu, Ni, Zn, and Mn on the growth and metal bioaccumulation of *Geobacillus toebii* subsp. *decanicus*, which showed the highest metal bioaccumulation for Zn (36.496 μg/g dry cell weight). This behavior indicates the potential of thermophilic microorganisms for the recovery of heavy metals from industrial wastewater, as in the case of *Geobacillus thermodenitrificans* used for bioremediation by the biosorption of As, Cr, and nitrate [110]. A total of 50 mg of dried cells of *Geobacillus thermantarcticus* was tested for the removal of Cd, Cu, Co, and Mn, achieving up to 85.4% for Cd removal, while *Anoxybacillus amylolyticus*, which was used in the same experimental setup, achieved 74.1% removal for the same metal [111]. Interestingly, Sundar et al. [112] explored the potential of biofilm formation using thermophiles. The biofilm produced by the consortium of *Bacillus subtilis* and *Bacillus cereus* was able to effectively remove 98% of Cr(III) from contaminated sand.

A HMs-tolerant bacterium, *Oceanobacillus profundus* KBZ 3-2 isolated from a polluted site, achieved a 97% and 54% removal of Pb (II) and (II) Zn, respectively [113]. *Bacillus* sp., isolated forms from heavy metal-contaminated environments, were combined to design a moving bed biofilm reactor, with the removal of Lead alone, or together with Cadmium and Nickel [114]. The detection of hazardous pollutants such as arsenic was achieved with the development of a biosensor exploiting the stability and adsorption capacity of *Thermus thermophilus* [115]. In addition, *Pseudomonas* sp. highlighted metal sequestration and an oxidation capacity. In particular, the halophilic and thermophilic *Pseudomonas* sp. 4-2 reached a Pb absorption of 226.24 mg/g for active cells at 55 °C and in the presence of a high concentration of NaCl [116]. Instead, *Pseudomonas aeruginosa* FZ-2, in the presence of a 10% NaCl concentration, was capable of increasing the affinity for Hg on the cell surface, which is crucial for directing the conversion of Hg^2+^ to Hg^0^ [117]. Recently, *Pseudomonas alcaliphila* NEWG-2 demonstrated a hexavalent chromium biosorption capacity of 97.2% at 50 mg/L and 96.1% at a saturated concentration of 200 mg/L [118]. This behavior suggests its applicability as a green technology for the treatment of Cr-contaminated water.

Table 2 lists the cited examples of extremophiles employed in biometallurgy.

#### 3.1.2. Organic Compounds

A variety of aliphatic, aromatic, and branched hydrocarbons and their derivatives, as well as several organic compounds, need to be degraded in the remediation of sites and water contaminated by crude oil and other anthropogenic activities. The different types of contaminants originate from a wide range of industrial, agricultural (pesticides and herbicides, etc.), and domestic activities, but there are also hydrocarbon pollutants from natural sources, such as reservoirs and volcanic processes in the deep sea [119]. These compounds are easily released into the air and soil and, although poorly soluble in water, they can be bioaccumulated, causing serious health effects in living organisms. The use of extremophiles can reduce both the time required for the remediation of marine and terrestrial ecosystems and dependence on aggressive chemical treatments. In this respect, thermophilic microorganisms make organic oil compounds more accessible for degradation in high-temperature environments [120].

Thermophilic bacteria, such as *Geobacillus* and *Bacillus* species, are potentially capable of degrading organics. *Geobacillus thermoleovorans* T80, in the treatment of hydrocarbon-contaminated soils, showed a hexadecane-degrading capacity at 60 °C of 71%, which improved further with the addition of rhamnolipid (biosurfactant), reaching 90% removal after 40 days [121]. *Geobacillus* and *Bacillus* strains, found on a volcanic Greek island, were able to grow on crude oil as the sole carbon source and degrade long-chain crude oil alkanes in the range of 46.6% to 87.7% [122]. *Geobacillus* spp. isolated from compost solubilized phenanthrene at 60 °C and degraded the PAH [123], while *Geobacillus stearothermophilus* A-2 isolated from a petroleum reservoir at 70 °C degraded PHA and long-chain n-alkanes up to 89.8% [124]. Moreover, *Bacillus licheniformis* removed up to 55% of crude oil, equivalent to a 3500 ppm crude oil concentration, from artificial seawater in hypersaline and alkaline environments (2.5 g/L NaCl and pH 8.5) [125]. Guevara-Luna et al. conducted studies on the biotransformation processes of benzo[a]pyrene by the thermophile *B. licheniformis* M2-7, producing phthalic acid as a less toxic intermediate [126].

The use of degradative microorganisms, and consequently the enzymes they synthesize, in many cases provides substrate-specific activity. Thus, complete degradation processes can often be significantly improved by using microbial consortia. Recently, *Bacillus megaterium*, *B. licheniformis*, *B. cereus*, and *Pseudomonas chlororaphis* isolated from soil contaminated by a pipeline leak can be used as tools for petroleum hydrocarbon degradation [127]. Two different strains, *B. licheniformis* ARMP2 and *P. aeruginosa* ARMP8, degraded oil products with an efficiency of about 88% and 73% in 48 h, respectively, with the potential to improve these results if used in consortia [128]. As another example, *Geobacillus jurassicus* and *Geobacillus subterraneus* from high-temperature oil fields grow selectively on benzoate but not on phenol, requiring the presence of their complementary activities for the complete degradation of crude oil [129]. Elumalai et al. [130] investigated the efficiency of *Geobacillus thermoparaffinivorans* IR2, *Geobacillus stearothermophillus* IR4, and *B. licheniformis* MN6 pure isolates and a consortium on the degradation of long-chain n-alkanes (C32 and C40). Biodegradation efficiencies were higher for C32 (90%) and C40 (87%) by the mixed consortium compared to the pure strains. Other thermophiles belonging to *Thermus* spp. have been exploited for the degradation of hydrocarbons. Two strains of *Thermus aquaticus* were successfully tested for benzene, toluene, ethylbenzene, and xylene degradation in a temperature range of 60–70 °C [131]. Over the same temperature range, and at a neutral pH, *Thermus brockii* efficiently broke down pyrene (40 mg h^−1^) and hexadecane (1000 mg h^−1^) [132]. The thermo-acidophile *S. solfataricus* is an archaeon capable of producing enzymes involved in the degradation of aromatics such as benzaldehyde and salicylate [133], and *S. solfataricus* 98/2 was able to degrade phenol through meta-ring cleavage at 80 °C and pH 3.2 [134].

Microorganisms from saline environments have also been investigated for their potential in hydrocarbon remediation, and also through their extracellular polymers, which act as biosurfactants, aggregating oils, and emulsifying hydrocarbons. The halophilic archaeon *Natrialba* sp. C21 could grow via the orthocleavage pathway using phenol, naphthalene, and pyrene as carbon sources at 25% (*w*/*v*) NaCl. This is useful for bioremediation in environments polluted by extreme levels of aromatic hydrocarbons [135].

Haloarchaea strains, isolated from hypersaline areas, were able to grow on a mixture of benzoic acid, p-hydroxybenzoic acid, and polycyclic aromatic hydrocarbons, even if the degradation mechanisms were not reported [136]. About 57% of hexadecane was degraded at a high saline concentration (3.6 M NaCl) within 24 days by *Halorientalis hydrocarbonoclasticus* sp. nov., which was isolated from a salt pan near an oil field (China) [137]. *Halobacillus* sp. EG1HP4QL was found to be able to utilize crude oil as the sole carbon source, also degrading aromatic hydrocarbons (51.2%), polycyclic aromatic hydrocarbons (43.5%), naphthalene (49.6%), paraffin (34.5%), and alcohol-benzene resins (25.5%) in the presence of heavy metals [138]. In a study by Gutierrez et al., *Halomonas* strain TG39, from an oil rig in the Gulf of Mexico, produced exopolysaccharides (EPS), which showed solubilizing properties for aromatic hydrocarbons and phenanthrene [139]. Enrichment with other EPS-producing bacteria (*Alteromonas*, *Colwellia* and *Pseudoalteromonas)* improved the prevention of oil aggregation in contaminated surface waters. *Marinobacter sedimentarum* and *M. flavimaris* bacteria were identified for their ability to hydrolyze aliphatic hydrocarbons (C9–C40), the aromatics benzene, biphenyl, phenanthrene, anthracene, and naphthalene [140], and decontaminate water microcosms without the use of any nitrogen fertilizers. The same behavior was also shown by *Marinobacter nanhaiticus* D15-8W, which is actively involved in degrading marine pollutants, including PAHs [141].

Among the extremophilic microorganisms, the cold-adapted ones have been shown to degrade petroleum hydrocarbons efficiently. *Oleispira antarctica* RB 8T, isolated from crude-oil enrichments of Antarctic seawater, was efficient in degrading hydrocarbons at 15 °C (oil waste, jet fuel, and crude oil), while for diesel, it performed best at 4 °C [142]. De Jesu et al. also confirmed the presence of bacterial and archaeal strains that degrade petroleum hydrocarbons in the Antarctic region [143]. The Arctic psychrotrophic *Pseudoalteromonas* sp. P29 preferentially biodegraded short-chain alkanes with an efficiency between 90 and 80% at 5 °C in a medium supplemented with mixed oil or vacuum oil [144]. *Pseudomonas* strain ST41 grew on the widest range of hydrocarbons at 4 °C and was able to degrade alkanes (C16/C20) with a 100% yield in 18 weeks [145]. *Pseudomonas* spp., in general, highlight a high level of tolerance to heavy metals, hydrocarbons, and other toxic compounds, which increases their suitability for use in bioremediation. At 7% NaCl (*w*/*v*) and pH 9, three haloalkaliphilic *Pseudomonas* strains demonstrated an important ability to degrade benzene, toluene, ethylbenzene, and xylene [146]. The addition of *P. aeruginosa* S5 to cooking wastewater was shown to be capable of reducing polycyclic aromatic hydrocarbons with a high molecular weight, from 9141.02 to 5117.16 µg/L, in 15 days [147]. A similar performance was achieved under dynamic cultivation by *Pseudomonas* strain AQNU-1, which exhibited a biodegradation efficiency of crude oil for n-alkanes (alkylcyclohexane, alkylbenzene, and alkyltoluene), with degradation ratios of 87–100% [148]. Hexadecane, nonadecane, fluorene, phenanthrene, and pyrene were degraded by the alkaliphilic, metallotolerant *P. aeruginosa* strain with an efficiency in the range of 40–80% in seven days [149]. The thermophilic strain AP02-1 of *P. aeruginosa*, capable of producing biosurfactants, reported high rates of hydrocarbon degradation in crude oil and diesel in the short term (7 days) at 45 °C [121]. In Table 3, we have listed the examples of extremophiles employed in organic remediation. The extensive literature concerning studies on extremophilic microorganisms and their ability to metabolize organic contaminants, therefore, highlights their effectiveness in bioremediation processes of polluted areas under extreme conditions.

#### 3.1.3. Radioactive Waste Treatment

The presence of radionuclides (e.g., Uranium, Thorium, and Radon) in soil and water represents a serious problem, also due to their low mobility and long decay times, which makes remediation difficult. These pollutants originate from mining, armament industries, and natural sources. Radioresistant microorganisms that have acquired specific mechanisms are able to survive in the presence of various radioactive pollutants by producing extremolytes. These secondary metabolites (betaine, melanin, trehalose, ectoin, etc.) are able to absorb a wide range of harmful radiation and protect the microorganism’s DNA from damage. Thanks to these properties, biomineralization, biosorption, and biotransformation processes can be exploited in the interaction between microorganisms and radioisotopes [75]. The solubility and mobilization of radionuclides within their deposits are influenced by the microbial presence due to environmental alteration (changes in pH, oxidation-reduction potential, etc.).

Numerous studies have investigated bioremediation using the poly-extremophilic bacteria *Deinococcus radiodurans* to remove radionuclides. *D. radiodurans* is one of the most radioresistant microorganisms and can live in extreme environments where ionizing radiation (up to 60 Gy/h) and ultraviolet rays are present, as well as withstanding oxidation and desiccation processes [150]. The recombinant *D. radiodurans*, capable of expressing a non-specific acid phosphatase in a high-radiation environment, was lyophilized and tested for radionuclide removal. The cells immobilized on polyacrylamide gels provided a removal of 70% for U(VI) and 90% for Cd [151]. In *D. radiodurans*, the alkaline phosphatase (PhoK) of *Sphingomonas* sp. was recombinantly expressed. DrPhoK cells showed 90% Uranium precipitation in the presence of liquid radioactive waste contamination [152]. In the presence of up to 6.4 kGy radiation, radioactive cobalt (^60^Co) was successfully removed (>60%) by the engineered *D. radiodurans* R1 [153]. The microorganism was also studied for its ability to remove radioactive iodine from contaminated water using silver particles that carried the bacterium [154]. The biogenic nanoparticles showed effective iodine removal of up to 99%, which can be used as a bioremediation platform. Another bacterium of the *Deinococcus* species isolated from arsenic-polluted water, *D. indicus* Wt/1a^T^, has the radiation resistance phenotype in addition to tested resistance to arsenate and arsenite [155]. Martínez-Rodriguez et al. studied Uranium biomineralization by *Microbacterium* sp. Be9, isolated from U-mill tailings [156], and found the microorganism capable of accumulating and precipitating U intracellularly in the presence of cellular phosphates.

The biomineralization/bioaccumulation to convert soluble Uranium radioisotopes into insoluble species was also investigated at the intracellular and extracellular level in *Shewanella putrefaciens* via c-type cytochrome activity. An extracellular reduction in U was also used by *Geobacter sulfurreducens* [157], suggesting that the methodologies are suitable for in situ bioremediation. In cell suspensions of *Geobacter metallireducens* GS-15 and *Shewanella oneidensis* MR-1 [158], a reduction in Uranium (VI), plutonium (Pu(VI), and Pu(V)) to the stable forms (U(IV) and Pu(IV)) was observed. In particular, Pu was precipitated in the form of nanoparticles on the surface of the bacteria or inside the cell walls. The pre-concentration of Uranium and Thorium prior to their evaluation in the environment has been developed using the thermophilic *B. cereus* as a new type of biosorbent for the determination of trace amounts of radionuclides [159]. Among the radioresistant microorganisms that can be used for the ecological management of radioactive waste are archaeal organisms. *Thermococcus gammatolerans*, a hyperthermophilic archaeon isolated from a hydrothermal vent and exposed to gamma rays, has several genes involved in the detoxification of harmful radiation products and shows high resistance to heavy metals like Cd, Co, and Zn [160].

Halophilic microorganisms *Halomonas* sp., *Halobacterium salinarum*, and *Halobacterium halobium* showed a good affinity for Europium(III) and Curium(III) absorption [161]. In contrast, *Halobacterium noricense* DSM-15987 and *Halobacterium* sp., isolated from the Waste Isolation Pilot Plant landfill, showed a typical multistage U(VI) bioassociation behavior. The first stage of biosorption involved phosphorylated and carboxylated groups in the complexation of U(VI), followed by biomineralization [161].

Although important from a bioremediation perspective, the microorganisms involved in this technique are often not cultivable, or they only work in consortia. Population analysis in groundwater contaminated with U(VI) and Fe (III) showed an association with the presence of *Geobacter* sp. in the treatment zone. The decrease in these microorganisms in the community was observed alongside a reduction in U and Fe, suggesting that optimizing the long-term maintenance of *Geobacter* could facilitate an in situ bioremediation strategy [162]. Table 4 lists the examples of extremophiles employed in radioactive waste treatment.

### 3.2. Plant Growth Promotion

Human activities and many environmental stressors, such as desertification, salinization, soil erosion, and heavy metal contamination, have a major impact on the progressive reduction in cultivable land [163]. Extremophiles capable of colonizing rhizosphere, phyllosphere, and internal plant tissues, known as plant growth-promoting bacteria (PGPB), play a key role in soil health by stimulating plant growth and adaptation under stress conditions through phytohormones and antimicrobial production, nutrient solubilization, and biological nitrogen fixation. Their use has proven to be a promising strategy for the development of sustainable agriculture [163].

The genus *Bacillus* in the rhizosphere is a PGPB that improves soil fertility and enriches the soil with macro and micronutrients. Kumari et al. [164] tested *P. aeruginosa* BHU B13-398 and *B. subtilis* BHU M against the pathogen *Rhizoctonia solani* in *Vigna radiata*. The BHU B13-398 strain increased the shoot and root length by 32.26% and 13.38%, respectively, while the BHU M strain increased these by 86.60% and 61.98%. Five strains of *Pseudomonas fluorescens*, used as biocontrol agents in black pepper against the pathogen *Phytophthora capsici*, also increased the root biomass by 30–135% due to the production of phytohormones (indolacetic acid-IAA and gibberellic acid-GA) and phosphorus solubilization [165]. Heat stress was tolerated by *B. cereus* SA1, with increases in shoot length (15.08%), root length (14.63%), fresh weight (27.28%), and dry weight (12.39%) in soybeans [166]. A reduction in abscisic acid (ABA) levels by three times and an increase in salicylic acid (SA) content from 30% to 53% were also observed. Similarly, *Bacillus* sp. PG-8 promoted seed germination of *Arachis hypogea* up to 70% with a seedling vigor index of 154, also producing IAA, GA, and ammonia exopolysaccharide [167]. *Pseudomonas* PTA-122608, found in the rhizosphere of an Antarctic grass, increased root growth by 110% after soybean inoculation and nodulation from *Bradyrhizobium japonicum* by 122%, with carbon and nitrogen accumulation by 64% and 55%, respectively [168].

In addition, PGPBs can help plants tolerate salinity stress by adjusting the K+/Na+ ratio. In this perspective, *B. licheniformis* HSW-16 increased the growth of wheat (root, sprout length, and weight) from 6% to 38%. The production of exopolysaccharides (EPS), osmolites, and the reduction in malondialdehyde (oxidative stress) resulted in a 51% decrease, with a simultaneous reduction in Na^+^ and an increase in K^+^ and Ca^2+^content [169]. Also, a rhizobial consortium of seven Bacilli improved the growth of cotton with an improvement of root length (17%), shoots (34%), chlorophyll content (34%), and leaf area (33%), also increasing K^+^ and reducing Na^+^ uptake [170]. At concentrations of 200–400 mM NaCl, the halophiles *Staphylococcus jettensis* F-11, *Zhihengliuella flava* F-9, *Bacillus megaterium* F-58, and *S. arlettae* F-71 were able to increase the root and shoot length and fresh weight of *Zea mays* L. [171]. The overexpression of the gene involved in plant growth (Glyma.03G226000) in halophilic bacteria *Stenotrophomonas* and *Exiguobacterium* was associated with a germination increase in soybean (35–43%) at 250 mM NaCl, doubling the length and dry biomass of roots [172].

Bacterial phytohormones can boost plant development in response to drought and salinity. For example, *Bacillus tequilensis* (SSB07) induced thermotolerance (35 °C) and improved the growth of cabbage seedlings via GA, IIA, and abscisic acid production [173]. Numerous rhizobacteria produce phytohormones under salt stress conditions, such as *B. subtilis*, which produces IIA under a wide range of pH conditions (5–9). *Bacillus thuringiensis* RZ2MS9 is also a producer of IAA [174] and can promote tomato growth, increasing the dry weight of sprouts by 24% and root length by 26%. *Pseudomonas aureantiaca* TSAU22 and *P. extremorientalis* TSAU6 and TSAU20 produce IAA and GA that attenuate seed dormancy induced by salt stress (100 mM NaCl) and promote root growth by up to 52% under stressed conditions [175].

Interestingly, reducing ethylene levels by degrading its precursor, 1-aminocyclopropane-1-carboxylic acid, due to PGPB, reduces physiological damage to plants and favors their growth in terms of height, root length, and leaf number. This ability has been observed in halotolerant strains from the *Achromobacter*, *Pseudomonas*, and *Rhizobium* genera [165]. Growth, resistance to pathogens, and environmental stress can be promoted by the bioactive compounds of bacteria. Increased antioxidant activities such as catalase (CAT), peroxidase, and superoxide dismutase (SOD) are essential to eliminate reactive oxygen species (ROS) that accumulate in response to stress. The toxic effects, due to high concentrations of Hg in *Lupinus albus*, were counterbalanced by CAT and SOD production by *Bacillus toyonensis* and *Pseudomonas syringe* that sustained plant growth [176]. The biomass of *Helianthus tuberosus* decreased upon exposure to Cd and Zn. However, growth was improved when it was treated with the endophytic strains *Pseudomonas* sp. 228 and 262 and *Serratia* sp. 246, which reduced oxidative stress. In the presence of Zn, the weight of sprouts increased by 145% and the weight of roots by 263% with the help of *Pseudomonas* sp. 228 [177]. Table 5 lists the examples of extremophiles employed in plant growth promotion.

### 3.3. Production of Biofuels and Biosurfactants

Extremophiles have great potential in lignocellulose bioprocessing and in bio-refinery research for the feasibility of this industry. As extensively reported, extremophiles and extremozymes are used in biofuel and high-value-added production from lignocellulose, with a reduced environmental impact compared to conventional processes, less energy, and high product yield. Considered among the best alternatives to fossil fuels, biofuels are derived from microbial activity on organic substrates. The first generation comes from food resources (e.g., sugarcane, corn, beetroot, wheat, and soya beans), while the second generation comes from non-food biomass, such as lignocellulosic residues (e.g., agricultural wastes, food industry, and household garbage) [178]. Despite significant progress, lignocellulose bio-refining remains a technological challenge that requires more effective pretreatment, new enzyme screening strategies, and process optimization.

Lignocellulose consists of cellulose, hemicellulose, and lignin. Cellulose (35–50%), a polysaccharide composed of linked glucose units, is the main constituent of lignocellulosic biomass. Hemicellulose (20–35%), a complex branched polysaccharide formed by different types of wood xylans, mannans, glucans, and xyloglucans, is closely associated with cellulose filaments and covalently bound to lignin. Lignin (5–30%) is an aromatic heteropolymer composed of ether and C-C bonds, which bind phenyl and aryl-C3 units. The component content changes in a single plant depending on age, growth stage, and several other conditions [133]. Extremophiles, capable of withstanding the drastic conditions required, play a key role as they act as natural biocatalysts in pretreatment, saccharification, and fermentation, in particular for bioethanol production [179]. In this context, lignocellulose can be decomposed by many microorganisms, including bacteria and archaea, thanks to their complex metabolic pathways. Countless microorganisms with (hemi)cellulolytic activity capable of catalyzing biomass hydrolysis in bioprocesses belong to the species *Alicyclobacillus*, *Anoxybacillus*, *Acidothermus*, *Bacillus*, *Caldanaerovirga*, *Caldicellulosirupto*, *Cellulomonas*, *Clostridium*, *Dictyoglomus*, *Geobacillus*, *Paenibacillus*, *Nesterenkonia*, *Paenibacillus*, *Pyrococcus*, *Rhodothermus*, *Sulfolobus*, and *Thermoanaerobacterium*, and *Thermotoga* [133]. Some interesting examples are given below.

Thermostable microorganisms, such as *Geobacillus* sp. R7, have been used for their ability to produce a cellulase hydrolyzed pretreated corn stover and cord grass at 70 °C, with 0.45–0.50 g ethanol/g glucose production after yeast fermentation and a glucose utilization efficiency of more than 99% [180]. The haloalkali-thermophile *Alkalilimnicola* sp. NM-DCM1 exhibited En5H cellulase activity (55 °C, pH 8.8, 2.5 M NaCl) specific for the hydrolysis of β-1,4 bonds and is also active on carboxymethylcellulose (CMC), Avicel, and cellobiose [181]. Using 110 U/g of En5H on rice straw increased the conversion of cellulose and hemicellulose by 28% compared to the control. *Anoxybacillus kamchatkensis* NASTPD13 expressed xylanases highly resistant to extreme pHs, denaturing agents, and organic solvents [182]; moreover, the β-xilosidase from *Anoxybacillus* sp. 3M improved the fermentable sugar yield in a brewery’s spent-grain bioconversion and catalyzed transxylosilation reactions in the presence of alcohols as acceptors [183]. The thermo-alkali-stable xylanase from *G. thermodenitrificans* A333, with maximum activity at 70 °C and resistance to metals and organic solvents, efficiently hydrolyzed agro-based biomass-producing xylooligosaccharides [184]. A cellulase/xylanase activity was expressed by *Chryseobacterium* genus working either on CMC or birchwood xylan [185]. The microbial consortium of *Ruminococcus* and *Clostridium* showed xylan degradation, and the addition of *Clostridium* sp. WST led to the conversion of xylan into bio-butanol (10.8 g/L), with an improvement of 98-fold. Moreover, on corn cob (xylan-rich biomass), a good yield of bio-butanol (1.09 g/L) was obtained [186].

Interestingly, a hyper-thermostable glycosidase produced by *Pyrococcus woesei* catalyzed the synthesis of 2-(β-galattosil)-ethyl methacrylate (Gal-EMA), with a product yield of 88%, which is useful for producing acrylic polymers functionalized with sugars [187].

Starch can also be used to produce biofuels; amylases from extremophiles, in particular *Bacillus* sp., are indicated here for α-amylase isolation. One example is the α-amylase produced by the thermophile *Bacillus* sp. NRC22017, which is capable of degrading starch as a cheap carbon source at 45 °C [188]. A promising strategy for improving the use of starchy biomass in biofuel production was represented by the use of α-amylase from *Bacillus megaterium* to degrade starch and produce hydrogen, ethanol, and succinic acid at 55 °C and an acidic pH of 4.5 [189].

Lignin, the most recalcitrant component of plant cell walls, can also be exploited efficiently, as demonstrated by the activity on alkaline lignin of *Bacillus ligniniphilus* L1 (50 °C), in which at least four kinds of lignin degradation pathways are present. After seven days of incubation at pH 9, the microorganism induced a 38.9% degradation of the lignin, producing useful aromatic compounds, including vanillic acid and vanillin [190]. Moreover, a small number of thermos- and halotolerant laccases were obtained from *Bacillus* sp. SS4 and *Thermobifida fusca*. These enzymes are able to depolymerize lignin into low-molecular-weight phenolics and aromatics, showing high potential in lignin valorization [179].

In addition to biofuel production, an emerging and promising area of extremophile use is biosurfactant synthesis. These bioactive compounds act as emulsifiers, reduce surface tension, and are used in a wide range of industries, including oil, detergents, cosmetics, and pharmaceuticals. The most effective producers are halophiles, thermophiles, and psychrophiles [191]. A relevant example is *Aneurinibacillus aneurinilyticus* SBP-11, isolated in the Gulf of Mannar (India), producing the lipopeptide biosurfactant, Aneurinifactin. This compound is stable across a wide range of pHs (2–9) and 80 °C, with a critical micellar concentration of 26 mg/L, and can reduce the surface tension of water. It also has high antimicrobial and oil-recovery activity and is concentration-dependent, making it suitable for biomedical use [192]. *Pseudomonas syringae* pv. *tabaci* also showed high biosurfactant production, primarily in the form of a mixture of syringopeptins and arthrofactins. The microbial extract reduced the surface tension of water to 36.89 mN/m, falling further to 29 mN/m in the presence of 5% *w*/*w* NaCl. The emulsion index reached 90% in coconut oil/water mixtures and maintained approximately 80% activity, even at 20% NaCl and 80 °C. The performance was comparable to or even better than that of commercial bioemulsifiers, such as xanthan, tara, and guar gum [193].

Although the use of extremophilic microorganisms is associated with some limitations, such as production costs and product quality, these can be overcome through advanced processes, such as high-temperature acetogenesis. In Table 6, we have listed extremophiles employed in biofuel production.

### 3.4. Medical Applications

Extremophilic microorganisms express specialized enzymes (extremozymes), osmoprotectants (extremolytes), and secondary metabolites that are naturally adapted to maintain activity and stability under conditions that typically denature conventional biomolecules. These characteristics make extremophiles highly promising for a wide range of medical applications, including diagnostics, drug development, therapeutic delivery, and vaccine technology [194].

One of the most impactful medical applications of extremophiles is in molecular diagnostics. The thermostable DNA polymerase Taq from *T. aquaticus* enabled the development of a polymerase chain reaction (PCR), a cornerstone of modern diagnostic and research technologies [6,195]. In addition, other extremozymes, such as ligases [196], reverse transcriptases [6], and helicases [197] derived from hyperthermophilic archaea and bacteria, have enhanced the robustness and speed of nucleic acid amplification techniques used in infectious disease diagnostics and genetic screening. Therapeutically, extremozymes offer advantages for enzyme replacement therapies, especially for disorders requiring enzyme activity in harsh environments, such as the gastrointestinal tract or inflammatory sites. Thermophilic aminoacylases from *Thermococcus litoralis* are being used to produce enantiomerically pure amino acids, which are vital in drug synthesis [198].

The production of robust bio-derived nanocarriers and stabilizers by extremophiles is also a key factor in drug delivery. Extremolytes such as cyclic di-phosphoglycerate (cDPG) and mannosylglycerate, found in hyperthermophilic archaea, act as natural osmoprotectants that stabilize therapeutic proteins and peptides during formulation and delivery. These molecules help to maintain protein folding and activity under oxidative stress, heat, or desiccation conditions often encountered in vivo [199,200,201]. Furthermore, extremophile-inspired liposomes and polymeric vesicles built to mimic archaeal membranes (made of ether-linked lipids) demonstrate excellent durability in acidic environments, which includes the stomach or tumor microenvironment. These systems are being explored for oral or tumor-targeted drug delivery, where conventional carriers degrade or lose functionality [202,203].

Moreover, the unique surface molecules and cell wall components of extremophilic archaea, such as glycosylated S-layer proteins and *archaeal* lipids, possess immunostimulatory properties that make them potential candidates as vaccine adjuvants. These molecules can engage innate immune receptors (e.g., Toll-like receptors), enhancing antigen presentation and cytokine production [204,205,206]. Studies have shown that lipid vesicles mimicking archaeal membranes can be formulated into *archaeosomes*, thermally and chemically stable adjuvants capable of inducing strong and long-lasting cellular and humoral immune responses [204]. Archaeosomes have shown promise in experimental vaccines against infections (e.g., *Mycobacterium tuberculosis*) and cancer, thanks to their ability to promote Th1-biased immune responses [206,207].

Extremophiles also synthesize antimicrobial peptides (AMPs), secondary metabolic products, and metal-chelating chemicals. Halophilic archaea such as *H. salinarum* produce halocins, antimicrobial peptides that inhibit other halophiles, and have potential as antibiotics [208]. Similarly, psychrophilic and acidophilic bacteria synthesize compounds with antioxidant and cytoprotective effects, which may support treatments for oxidative stress-related conditions [209,210,211]. The radiation-resistant bacterium *D. radiodurans* is under investigation for its unique DNA repair systems and antioxidants, offering potential for radioprotective therapies during cancer radiotherapy or in environments with high oxidative damage [212].

Extremophilic enzymes and proteins are increasingly integrated into biosensor technologies for point-of-care diagnostics, owing to their exceptional stability, activity, and substrate specificity [213]. For instance, thermophilic glucose-6-phosphate dehydrogenases or oxidases derived from *Thermoplasma* or *Sulfolobus* spp. can be immobilized on electrochemical platforms to detect glucose or other metabolites under a broad range of temperatures and pH levels [214,215]. Additionally, a thermostable asparaginase from *Archaeoglobus fulgidus* has been utilized in the development of an asparagine biosensor, demonstrating enhanced stability compared to its mesophilic counterparts [216]. Similarly, a thermostable glucose-6-phosphate dehydrogenase from *Aquifex aeolicus* has been employed in high-temperature amperometric biosensors, maintaining functionality at temperatures (80 °C), where traditional enzymes fail [217].

Moreover, archaeal membrane lipids are increasingly employed as biomimetic platforms in biosensor technologies due to their exceptional stability under extreme conditions [218]. For instance, liposomes derived from the polar lipid fraction E (PLFE) of *S. acidocaldarius* demonstrate low proton permeability and maintain structural integrity across a broad temperature range (60–90 °C) [219]. Such properties are particularly advantageous for developing biosensors intended for diagnostics in resource-limited settings, space missions, and hostile physiological niches like acidic tumors or wound exudates, where conventional lipid-based systems may fail [220]. Therefore, the integration of archaeal lipids into biosensor platforms enhances their durability and functionality, paving the way for reliable diagnostics in challenging conditions.

Interestingly, extremophilic microorganisms are also used in synthesis and organic transformations thanks to the biocatalysts that can be isolated from them. The alkalophile *Rhodococcus* sp. strain ECU0066 was able to produce a sulfide monooxygenase with enantioselectivity towards sulfides. This indicates good potential for application in the asymmetric synthesis of sulfide enantiopure compounds, which are valuable tools in the synthesis of chiral and complex molecules, with a significant impact in various areas of chemistry and pharmaceuticals [221].

Of great importance was the discovery of CRISPR-Cas systems in extremophilic archaea, which has transformed genome editing. Originally described in halophiles like *Haloferax volcanii*, these systems have been adapted into powerful tools for therapeutic gene-editing [222]. Ongoing research aims to harness the diverse CRISPR-Cas variants from extremophiles for more precise, efficient, and temperature-tolerant genome engineering applications, including gene therapy and personalized medicine [223,224].

Table 7 lists examples of extremophiles employed in medical applications.

### 3.5. Nanoparticles Production

The extraordinary adaptability of extremophilic microorganisms not only enables the production of resilient enzymes, proteins, and bioactive compounds with therapeutic potential but also opens new frontiers in nanomedicine. On the basis of their established roles in diagnostics, drug development, and biosensing, extremophiles are now recognized as promising biological factories for the sustainable synthesis of nanoparticles (NPs)—a rapidly growing area of application within medical biotechnology. Unlike conventional physical and chemical methods of nanoparticle synthesis, which often involve harsh reagents and energy-intensive processes, extremophiles utilize their native enzymatic machinery and redox capabilities to produce metal and metal oxide nanoparticles under environmentally friendly conditions [225]. The biologically synthesized nanoparticles inherit the stability, functionality, and biocompatibility characteristics of extremophile-derived biomolecules, making them particularly suited for use in drug delivery systems, biosensors, imaging, vaccine platforms, and antioxidant therapies areas previously highlighted in their soluble molecular counterparts [194].

Extremophiles synthesize nanoparticles via enzymatic reduction in metal ions, the secretion of redox-active metabolites, or biomineralization on the cell surface. These processes may occur intracellularly or extracellularly and are often mediated by specific extremozymes or membrane-bound reductases. Microbial proteins or polysaccharides, resulting in highly stable, functional, and biocompatible nanomaterials [226,227], typically stabilize the nanoparticles produced.

Thermophilic bacteria such as *Geobacillus* sp. ID17 demonstrated the ability to biosynthesize gold nanoparticles (AuNPs) through NADH-dependent reductase enzymes [225]. These enzymes facilitate the reduction in Au^3+^ to elemental gold, with the nanoparticles predominantly forming intracellularly and exhibiting quasi-hexagonal shapes ranging from 5 to 50 nm [228]. The process is enzymatically driven, as evidenced by the significant decrease in activity upon treatment with protein-denaturing agents. Halophilic archaea, such as *H. volcanii*, have been employed in the production of silver and gold nanoparticles [229]. These organisms can tolerate high salt concentrations and manage metal toxicity, enabling them to reduce metal ions and form nanoparticles [230]. The biosynthesis process is eco-friendly and leverages the natural metabolic pathways of extremophiles. Microorganisms adapted to acidic or alkaline environments possess enzymes that remain active under extreme pH conditions and assist in the reduction and precipitation of metal ions, resulting in nanoparticle production [225]. For instance, certain acidophilic and alkaliphilic bacteria have been reported to synthesize metal NPs, although specific mechanisms and examples are less documented compared to thermophiles and halophiles [231]. Nanoparticles synthesized by extremophiles have emerged as valuable biofactories with unique physico-chemical properties, rendering them highly suitable for advanced drug delivery applications. These NPs resist aggregation in saline or oxidative environments, which is crucial for maintaining dispersion during storage and ensuring bioavailability in vivo [225]. Below are notable examples illustrating the potential of extremophile-derived NPs in drug delivery systems.

Halophilic archaea such as *H. volcanii* and *Halococcus salifodinae* and thermophilic bacteria like *Geobacillus* spp. and *T. thermophilus* have proven to be capable of producing gold and silver nanoparticles (AuNPs and AgNPs) through biosynthesis [229,232,233,234,235]. These organisms use their native biomolecules (e.g., enzymes, peptides, and membrane components) to reduce metal ions and stabilize the resulting nanoparticles. The metal NPs produced are typically small, uniformly dispersed, and functionalized with biologically active groups, making them ideal for surface conjugation with therapeutic agents [236]. The radioresistant bacterium *D. radiodurans* has been employed to biosynthesize gold nanoparticles (AuNPs) through the reduction in Au(III) ions. These biogenic AuNPs exhibit diverse morphologies, including spherical and triangular shapes, with an average size of approximately 43.75 nm. NPs are stabilized by capping them with native proteins and biomolecules, enhancing their biocompatibility and making them promising candidates for drug delivery applications [237]. The hypersaline marine bacterium *Mesoflavibacter zeaxanthinifaciens* produces extracellular polymeric substances (EPS) that can be used to synthesize silver nanoparticles (AgNPs) without the need for chemical-reducing agents. The biogenic AgNPs have demonstrated significant antibacterial and antibiofilm activities, suggesting their use as drug delivery agents targeting bacterial infections [238].

Some extremophile-derived NPs exhibit pH-responsiveness, allowing for controlled drug release in acidic environments such as tumors. The thermophilic *Geobacillus wiegelii* strain GWE1 has demonstrated the ability to biosynthesize elemental tellurium and selenium NPs. By adjusting culture conditions such as pH and temperature, it was possible to control the NP’s size and shape. Such control is crucial for tailoring nanoparticles for specific drug delivery applications [225].

Extremophile-synthesized nanoparticles serve as both antigen carriers and immunostimulatory adjuvants. These extremophile-derived NPs offer advantages such as structural stability under harsh conditions and the ability to elicit robust immune responses, making them promising candidates for next-generation vaccine delivery systems [239]. For instance, gas vesicle nanoparticles (GVNPs) from *Halobacterium* sp. NRC-1 have been engineered to display antigens from various pathogens with efficacy in reducing bacterial burden in mice when used in conjunction with live attenuated *Salmonella* vaccines [240]. Similarly, GVNPs displaying *Chlamydia trachomatis* antigens have been shown to engage Toll-like receptors and stimulate pro-inflammatory cytokine production in vitro, indicating their potential as vaccine carriers [241]. Archaeaosomes, lipid-based vesicles derived from archaea such as *Halorubrum tebenquichense*, have also been utilized as vaccine adjuvants. These archaeosomes have been effective in eliciting immune responses against pathogens like *Trypanosoma cruzi* and *Listeria monocytogenes* in animal models [242]. Furthermore, the radioresistant *D. radiodurans* has been employed to produce AuNPs that can be functionalized for antigen delivery, enhancing immune responses in vaccine formulations [237]. NPs produced by halophilic archaea are being evaluated for nasal and transdermal vaccine delivery, given their ability to penetrate mucosal barriers and remain stable under physiological stress [243]. These examples underscore the potential of extremophile-derived nanoparticles in developing robust, eco-friendly vaccine adjuvants and carriers.

The growing interest in extremophile NPs also finds application in biosensor development and diagnostic tools, particularly in electrochemical and optical biosensing. For instance, the halophilic archaeon *Halococcus salifodinae* BK3 produces tellurium NPs exhibiting antibacterial activity, which can be integrated into biosensor platforms for pathogen detection [234]. Similarly, *Halobacillus* sp. DS2 synthesizes Cd sulfide quantum dots under high-salinity conditions, yielding fluorescent that are NPs stable in saline environments, which is ideal for optical biosensors [244]. Additionally, *Alkalibacillus* sp. w7, isolated from a salt lake, generates zinc oxide NPs with notable photocatalytic and antimicrobial properties, enhancing biosensor sensitivity and functionality [245]. Gold and silver NPs from thermophiles provide high surface plasmon resonance and conductivity, improving signal sensitivity [246]. They are used in glucose, cancer marker, and pathogen detection platforms.

NPs synthesized by extremophiles also exhibit notable antioxidant and cytoprotective properties, offering promising applications in biomedical fields. These NPs can scavenge reactive oxygen species (ROS) and reduce cellular oxidative stress, showing potential in therapies targeting neurodegeneration, wound healing, and inflammation. For instance, extremophilic *Actinobacteria* isolated from magnesite-rich soils have been utilized to synthesize magnesium oxide nanoparticles (MgO NPs). These biogenic MgO NPs demonstrated significant antioxidant activity and in vivo compatibility, which are useful as cytoprotective agents in therapeutic applications [247]. Moreover, the cyanobacterium *Desertifilum* sp. TN-15 was employed to biosynthesize zinc oxide nanoparticles (ZnO NPs) and highlighted significant antioxidant activity, as evidenced by their free radical scavenging capabilities. Additionally, they exhibited antihemolytic effects on red blood cells and low cytotoxicity, which is useful for biomedical applications [248]. *Acinetobacter* sp. GWRVA25 was utilized to biosynthesize gold nanoparticles (AuNPs), which displayed notable antioxidant activity, which is beneficial for potential cytoprotective agents [249]. There is also evidence that *Bacillus paramycoides* can produce selenium-based NPs with strong antioxidant capacity [250]. Furthermore, bacterioruberin from *H. tebenquichense*, combined with dexamethasone in archaeal lipid-based NPs, showed strong antioxidant and anti-inflammatory effects. These ultra-small, macrophage-targeted NPs effectively restored intestinal barrier integrity in an inflammation model [251]. Additionally, *D. radiodurans*, renowned for its resistance to ionizing radiation, accumulates manganese complexes that protect proteins from oxidative damage. This mechanism contributes to its remarkable resilience and suggests potential applications of its manganese-based systems in developing antioxidant therapies [237].

Nanoparticles synthesized by extremophiles are also emerging as promising agents for antibacterial applications. For instance, AgNPs produced by the thermophilic *Geobacillus* spp. have demonstrated potent antibacterial activity against both *E. coli* and *Staphylococcus aureus*, primarily through membrane disruption and reactive oxygen species (ROS) generation [233]. Moreover, AuNPs biosynthesized by *D. radiodurans* have shown effective antibacterial activity with significant inhibitory effects on both Gram-positive and Gram-negative bacteria. This is achieved by inducing membrane damage and increasing oxidative stress, thus compromising bacterial viability [252]. In addition to silver-based systems, selenium nanoparticles (SeNPs) synthesized using halophilic bacteria have also demonstrated effective antibacterial activity. For example, *Haloferax*-derived SeNPs significantly inhibited the growth of *P. aeruginosa* by inducing oxidative stress and compromising membrane integrity [253]. These extremophile-derived nanoparticles have found applications in coating biomedical devices to prevent biofilm formation, incorporation into wound dressings to promote healing and prevent infection, and development in antimicrobial packaging materials for food preservation. Their ability to inhibit multidrug-resistant bacteria highlights their potential as alternatives to conventional antibiotics in clinical and environmental settings [227].

Table 8 summarizes the production and utilization of several NPs reported.

## 4. Conclusions

Extremophilic microorganisms, with their ability to live and adapt to some of the most inhospitable ecological niches on Earth, provide the opportunity to study the complex and attractive capabilities and mechanisms that enable them to overcome the limitations of life in extreme environments. The evolutionary processes that enable microorganism adaptation to their challenging ecosystems have endowed extremophiles and their molecular constituents with unique properties that perfectly support their vital functions in hostile conditions. The peculiar adaptive characteristics allow the use of these microorganisms and their components to solve many actual challenges in a sustainable way through their exploitation in several processes that require harsh conditions. These processes include waste and radioactive residue management, bioremediation and sustainable agriculture improvement, and the recovery of valuable bioactive molecules for use in bioenergy, biomedical applications, and, interestingly, nanoparticle production. The idea of using extremophilic biocatalysts as an alternative to conventional methods has become crucial for sustainable, efficient, and eco-friendly developments needed for the transition to a green economy. Research on extremophiles and their extremozymes is increasingly driven by the need to achieve the 17 Sustainable Development Goals (SDGs) set out in the United Nations’ 2030 Agenda for Sustainable Development [7]. Their unique biocatalytic capabilities are pivotal for developing sustainable biotechnological solutions in several fields, such as advancing sustainable bioenergy, enabling innovative and sustainable industrial processes crucial for a circular economy, enhancing solutions for environmental remediation and climate change mitigation, and contributing to improving strategies to achieve good health and well-being. It is essential to address current technical and knowledge gaps in order to unlock the full potential of extremophilic microorganisms and extremozymes that contribute to the transformative objectives of the SDGs. One of the major bottlenecks in the advancement of extremophiles and extremozymes is the cost of production and the development of consolidated processes on a large scale. From this standpoint, further in-depth studies are required to uncover the mechanisms of extremophilic life and ways to improve them, also through genetic engineering. This would allow for the increase in the yield of their products and metabolites, the improvement and widening of biotechnological, environmental, and medical applications, and the circumventing of any potential side effects in their employment. Extremophiles lie at the intersection of environmental science, biotechnology, and our understanding of life on Earth. Given the unlimited potential of life under extreme conditions, the ongoing evolution of research in this area significantly contributes to many different fields.

## Figures and Tables

**Table 1:** Extremophiles cited in Section 2 and their growth conditions.

Groups of Microorganisms	Species	Growth Conditions	Ref.
Thermophiles	*Methanocaldococcus jannaschii*	85 °C, 20 Mpa	[9]
	*Methanopyrus kandleri*	122 °C, 20 MPa	[10,11]
	*Geogemma barossii* strain 121	121 °C	[12]
	*Pyrococcus furiosus*	100 °C, 0.1 MPa	[13]
	*Pyrococcus horikoshii*	100 °C, 15 MPa	[14]
	*Pyrococcus abyssii*	100 °C, 20 MPa	[15]
	*Aquifex aeolicus*	85–95 °C, pH 5.4–7.5	[16]
	*Aquifex pyrophilus*	95 °C, pH 5.4–7.5	[16]
	*Saccharolobus solfataricus* (formerly *Sulfolobus solfataricus*)	80 °C, pH 2.0–4.0	[17]
	*Sulfolobus acidocaldarius*	80 °C, pH 2.0	[17]
	*Sulfurisphaera tokodaii* (formerly *Sulfolobus tokodaii*)	80 °C, pH 2.0–3.0	[18]
	*Saccharolobus islandicus* (formerly *Sulfolobus islandicus*)	75–80 °C, pH 2.0–3.0	[17]
	*Thermotoga maritima*	65–80 °C, pH 7.0	[19]
	*Thermotoga neapolitana*	65–80 °C, pH 7.0	[20]
Psycrophiles	*Psychromonas ingrahamii*	−12 °C	[21]
	*Planococcus halocryophilus* Or1	−15 °C	[22]
	*Shewanella benthica*	4–10 °C, 50 MPa	[23]
	*Colwellia hadaliensis*	2 °C, 70 MPa	[24]
	*Moritella yayanosii*	4 °C, 100 MPa	[25]
	*Micrococcus cryophilus*	20–26 °C	[26,27]
	*Rhodococcus erythropolis*	4–37 °C	[26,27]
	*Marinomonas protea*	4–30 °C	[26,27]
	*Marinomonas primoryensis*	5–30 °C	[26,27]
Acidophiles	*Acidithiobacillus thiooxidans*	pH 2.0–3.0	[28]
	*Acidithiobacillus caldus*	pH 2.0–2.5	[29]
	*Acidithiobacillus ferroxidans*	pH 2.0	[30]
	*Leptospirillum ferrooxidans*	pH 1.0–2.0	[31]
	*Picrophilus torridus*	pH 0.7, 60 °C	[32,33]
	*Picrophilus oshimae*	pH 0.0, 65 °C	[32]
	*Sulfolobus acidocaldarius*	pH 2.0, 80 °C	[17]
	*Saccharolobus solfataricus* (formerly *Sulfolobus solfataricus*)	pH 2.0–4.0, 80 °C	[17]
	*Acidianus brierleyi*	pH 1.2–2.0, 70 °C	[34]
	*Acidianus infernus*	pH 2.0, 96 °C	[34]
	*Metallosphaera sedula*	pH 2.0, 75 °C	[34]
	*Thiobacillus thiooxidans*	pH 2.0–3.0	[35]
Alkaliphiles	*Bacillus alkaliphilus*	pH 9.5–10.5	[36]
	*Alteribacter natronophilus * (formerly *Bacillus natronophilus*)	pH 8.0–12.0	[37]
	*Natronospira bacteriovora*	pH 8.0–10.4	[38]
	*Desulfonatronovibrio hydrogenovorans*	pH 9.5–10.0, 3% NaCl	[39]
	*Halorhodospira halophila*	pH 9.0, 13% NaCl	[40]
	*Desulfonatronum lacustre*	pH 9.0, 0.2% NaCl	[41]
	*Desulfonatronum thiodismutans*	pH 8.0–10.0, 1.7% NaCl	[41]
	*Sporosarcina pasteurii* (*formerly Bacillus pasteurii*)	pH 7.0–9.0	[38]
	*Bacillus halodurans*	pH 10.0–10.5	[42]
	*Marinospirillum alkaliphilum*	pH 7.0–11.0, 0.2–5% NaCl	[43]
	*Nitrincola tapanii*	pH 8.0–10.5, 1% NaCl	[44]
	*Limnospira fusiformis * (formerly *Arthrospira fusiformis*)	pH 8.0–10.0	[45]
	*Halorhodospira halochloris*	pH 8.0–9.0, 14–27% NaCl, 50 °C	[46]
	*Desulfonatronum zhilinae*	pH 8.0–10.5, 0.5–10% NaCl	[47]
	*Halonatronomonas betaini*	pH 7.0–10.0, 6–17.5% NaCl	[48]
Halophiles	*Halomonas elongata*	10–16% NaCl	[49]
	*Methylarcula marina*	3–6% NaCl, pH 7.5–8.5	[49]
	*Methylarcula terricola*	3–6% NaCl	[49]
	*Spiribacter salinus*	6% NaCl, pH 7.0–9.0	[50]
	*Chromohalobacter salexigens*	3–20% NaCl	[50]

**Table 2:** Examples of extremophiles employed in biometallurgy.

Microorganisms	Process	Heavy Metals	Ref.
*Acidithiobacillus ferrooxidans*	Biomining Bioleaching	Cr, Co, Cu, Li, Mn, Ni, Pb, U, Zn	[101,102]
*Acidithiobacillus thiooxidans*	Bioleaching	Cd, Cu, Cr, Ni, Pb, Pu, Zn	[28]
Consortium of *A. thiooxidans*, *A. ferrooxidans* and *L. ferrooxidans* and heterotrophic bacteria Fe/S-oxidizing	Bioaugmentation	Cu, Cd, Hg and Zn	[31]
*Acidocella aromatica*	Biosorption	V	[103]
*Acidiphilium symbioticum*	Biosorption	Cd	[104]
*Halomonas smyrnensis* KS802	Bioreduction	Cr	[105]
*Sulfobacillus thermotolerans*	Biosorption	Zn	[106]
*Sulfobacillus thermosulfidooxidans*	Biosorption	Cd, Cu, Ni, Zn	[107]
*Thermus scotoductus*	Biosorption	Eu	[108]
*Geobacillus toebii* subsp. *decanicus*	Bioaccumulation	Cd, Cu, Mn, Ni, Zn	[109]
*Geobacillus thermodenitrificans*	Biosorption	As, Cr	[110]
*Geobacillus thermantarcticus*—*Anoxybacillus amylolyticus*	Biosorption	Cd, Co, Cu, Mn	[111]
Consotium of *B. subtilis*–*B. cereus*–*Bacillus* sp.	Bioremoval Biosorption	Cr, Cd, Ni, Pb	[112,113,114]
*Oceanobacillus profundus* KBZ 3-2	Biosorption	Pb, Zn	[113]
*Thermus thermophilus*	Biosensing	As	[115]
*Pseudomonas* sp. 4-2	Biosorption	Pb	[116]
*Pseudomonas aeruginosa* FZ-2	Biosorption	Hg	[117]
*Pseudomonas alcaliphila* NEWG-2	Biosorption	Cr	[118]

**Table 3:** Examples of extremophiles employed in organic compounds remediation.

Microorganism	Conditions	Organic Target	Ref.
*Geobacillus thermoleovorans* T80	60 °C—pH 7.0	Hexadecane	[121]
*Geobacillus* sp.—*Bacillus* sp.	60–80 °C—pH 7.8	crude oil, phenanthrene, PAH	[122,123]
*Geobacillus stearothermophilus* A-2	70 °C—pH 7.0	PHA, n-alkanes	[124]
*Geobacillus jurassicus*–*Geobacillus subterraneus*	55–60 °C—pH 7.0	Crude oil, phenols	[129]
Consortium of *G. thermoparaffinivorans* IR2, *G. stearothermophillus* IR4 and *B. licheniformis*	50 °C—pH 7.0	long-chain n-alkanes	[130]
*Bacillus licheniformis*	37 °C—pH 8.5—2.5 g/L NaCl	crude oil, benzo[a]pyrene	[125,126]
*Pseudoalteromonas* sp. P29	5 °C—pH 7.0–8.0—1–5% NaCl	short-chain alkanes	[144]
*Pseudomonas* strain ST41	4 °C—pH 7.5	hydrocarbons alkanes	[145]
*P. aeruginosa* S5	30 °C—pH 7.5	PHA	[147]
*P. aeruginosa* san ai	30 °C—pH 8.0–9.0	Hexadecane, nonadecane, fluorene, phenanthrene, and pyrene	[149]
Consortium of *B. megaterium*, *B. licheniformis*, *B. cereus*, *P. chlororaphis* Consortium *of B. licheniformis* ARMP2 and *P. aeruginosa* ARMP8	25 °C—pH 7.25 30–40 °C—pH 7.0–9.0	Hydrocarbon	[127,128]
*Thermus aquaticus*	60–70 °C—pH 7.5	benzene, toluene, ethylbenzene, xylene	[131]
*Thermus brockii*	60–70 °C—pH 6.5	Pyrene–hexadecane	[132]
*S. solfataricus*	80 °C—pH 3.2	Benzaldehyde, salicylate, phenols	[133,134]
*Natrialba* sp. C21	40 °C—pH 3.0—25% NaCl	PHA	[135]
*Halorientalis hydrocarbonoclasticus* sp.,	37 °C—pH 7.0—3.6 M NaCl	hexadecane	[137]
*Halobacillus* sp. EG1HP4QL	35 °C—pH 8.0—5% NaCl	aromatic hydrocarbons, PHA, naphthalene, paraffin, alcohol-benzene resins	[138]
*Halomonas* strain TG39	28 °C—pH 8.0	PHA-phenanthrene	[139]
*M. sedimentarum, M. flavimaris, M. nanhaiticus* D15-8W	30 °C—pH 7.0—1–1.5 M NaCl	aliphatic hydrocarbons, benzene, phenanthrene, anthracene naphthalene PHA	[140,141]
*Oleispira antarctica* RB 8T	4–15 °C—pH 7.0	Hydrocarbon	[142]

**Table 4:** Examples of extremophiles employed in radioactive waste treatment.

Microorganisms	Sources	Process	Heavy Metals	Ref.
*Deinococcus radiodurans*	Terrestrial and aquatic environments.	Bioprecipitation Bioleaching	Cd, Co, I, U	[151,152,153,154]
*Deinococcus indicus* Wt/1a^T^	Arsenic-polluted water	Bioleaching	As(III), As(V)	[155]
*Microbacterium* sp. Be9	U-mill tailings	Biomineralization	U	[156]
*Shewanella putrefaciens* *Geobacter sulfurreducens*	Water sources, natural gas and petroleum reserves	Biomineralization bioaccumulation	U	[157]
*Geobacter metallireducens* GS-15 *Shewanella oneidensis* MR-1	sediments of frozen Lakes, freshwater sediments	Bioprecipitation	Pu, U	[158]
*Bacillus cereus*	Soil, vegetation	Biosorption	Th, U	[159]
*Halomonas* sp., *Halobacterium salinarum*, *Halobacterium halobium*	Rock salt	Biosorption	Eu,Cu	[160]
*Halobacterium noricense* DSM-15987 *Halobacterium* sp.	Waste Isolation Pilot Plant landfill	Biosorption Biomineralization	U(VI)	[161]
*Geobacter* sp.	Contaminated groundwater	Biomineralization bioaccumulation	U(VI) and Fe (III)	[162]

**Table 5:** Extremophiles employed in plant growth promotion.

Biotic/Abiotic Stress	Extremophiles	Crops	Action	[Ref.]
Plant pathogens	*P. aeruginosa* BHU-B13-398 *B. subtilis* BHU M *P. fluorescens*	*Vigna radiata* Black pepper	P solubilization, ammonia, siderophore, HCN, IAA and GA production	[164,165]
Heat stress	*Bacillus cereus* SA1 *Bacillus* sp. PG-8 *Pseudomonas* PTA-122608, *Bacillus tequilensis* (SSB07)	Soybean *Arachis hypogea*, Cabbage seedlings	metabolites production (GA, IAA, ABS) plant–microorganism interactions	[166,167,168,173]
Salinity	*B. licheniformis* HSW-16, *Bacilli* spp., *S. jettensis* F-11, *Z. flava* F-9, *B. megaterium* F-58, *S. arlettae* F-71, *Stenotrophomonas*, *Exiguobacterium* sp., *P. aureantiaca* TSAU22, *P. extremorientalis* TSAU6—TSAU20, *Achromobacter*, *Pseudomonas*, *Rhizobium* sp.	Wheat, Cotton, *Zea mays* L, Soybean	regulation of the K+/Na+ ratio; production of EPS, ACC-deaminase activity; P solubilization; biofilm formation, up-regulation of growth gene, IAA and GA production, ACC-deaminase activity	[165,169,170,171,172,175]
Drought and salinity	*Bacillus thuringiensis* RZ2MS9	Tomato	IAA	[174]
Heavy metals	*B. toyonensis*, *P. syringae Pseudomonas* sp. 228–262, *Serratia* sp. 246	*Lupinus albus Helianthus tuberosus*	ACC deaminase, CAT and SOD activity, IAA production	[176,177]

**Table 6:** Extremophiles employed in biofuels production.

Extremophiles	Carbon Source	Enzymes	Applications	[Ref.]
*Geobacillus* sp. R7	Corn stover, Cord grass	Cellulase	Bioethanol	[180]
*Alkalilimnicola* sp. NM-DCM1	Rice straw, CMC, Avicel, cellobiose	Cellulase	saccharification of lignocellulosic material	[181]
*Anoxybacillus kamchatkensis*	birchwood xylan	Xylanase	Hemicellulose saccharification, pulping.	[182]
*Anoxybacillus* sp. 3M	BSG	β-xylosidase	Food, pharmaceutical, Bioethanol	[183]
*Geobacillus thermodenitrificans* A333	BSG, corn cobs, grape cane, wheat straw	Xylanase	Xylooligosaccharides, Bioethanol	[184]
*Chryseobacterium genus*	straw and spent mushroom	Cellulase/xylanase	Biofuel	[185]
*Ruminococcus* sp., *Clostridium* sp.	Corn cob	Hemicellulolytics enzymes	Bio-butanol	[186]
*Pyrococcus woesei*	Lactose	Glycosidase	Acrylic polymers	[187]
*Bacillus* sp. NRC22017	Starch	α-amylase	brewing industry	[188]
*Bacillus megaterium*	Starch	α-amylase	Biohydrogen, bioethanol, succinic acid	[189]

**Table 7:** Extremophiles and extremozymes employed in medical application.

Application Area	Key Extremophile-Derived Components	Source Organism/Type	Medical Relevance	Ref.
Molecular Diagnostics and Enzyme Therapy	Taq DNA polymerase, ligases, reverse transcriptases, helicases	*Thermus aquaticus*, *hyperthermophilic archaea*	PCR and nucleic acid amplification for diagnostics and genetic testing	[6,195,196,197]
	Thermophilic aminoacylases	*Thermococcus litoralis*	Production of enantiopure amino acids for drug synthesis	[198]
Drug Delivery	Extremolytes (cDPG, mannosylglycerate)	*Hyperthermophilic archaea*	Stabilize proteins/peptides during formulation and delivery	[199,200,201]
	Liposomes mimicking archaeal membranes (ether-linked lipids)	*Archaea *(e.g., *Sulfolobus* spp.)	Oral and tumor-targeted drug delivery in acidic or oxidative environments	[202,203]
Vaccine Adjuvants	Glycosylated S-layer proteins, archaeal lipids archaeosomes	*Archaea*	Immune activation via TLRs; enhance antigen presentation; experimental vaccines	[204,205,206,207]
Antimicrobial/Antioxidant/Radioprotective	Halocins (antimicrobial peptides)	*Halobacterium salinarum*	Novel antimicrobial agents	[208]
	Antioxidant compounds	*Psychrophilic*, *acidophilic bacteria*	Cytoprotection in oxidative stress conditions	[209,210,211]
	DNA repair enzymes, radioprotective molecules	*Deinococcus radiodurans*	Radioprotection in cancer therapy and high-radiation environments	[212]
Biosensors	Thermostable oxidases, dehydrogenases (e.g., G6PDH)	*Thermoplasma*, *Sulfolobus*, *Aquifex aeolicus*	Electrochemical biosensors for metabolites under extreme pH/temperature	[214,215,217]
	Thermostable asparaginase	*Archaeoglobus fulgidus*	High-temperature biosensing of amino acids	[216]
	Archaeal membrane lipids (e.g., PLFE)	*Sulfolobus acidocaldarius*	Biomimetic platforms for biosensors in hostile or resource-limited environments	[219]
Genome Editing	CRISPR-Cas systems	*Halophiles *(e.g., *Haloferax volcanii*)	Tools for therapeutic genome editing and synthetic biology applications	[222]

**Table 8:** Extremophiles in nanoparticle production.

Application Area	Extremophiles Involved	Type of NPs	Key Features/Mechanisms	Ref.
Nanoparticle Biosynthesis	*Geobacillus* sp., *Haloferax volcanii*, acidophilic/alkaliphilic bacteria	AuNPs, AgNPs, SeNPs, TeNPs	Enzymatic reduction, redox-active metabolites, biomineralization, stable and eco-friendly	[225,227,228,229,230,231]
Drug Delivery	*Geobacillus* spp., *Deinococcus radiodurans*, *Mesoflavibacter zeaxanthinifaciens*	AuNPs, AgNPs, SeNPs	Small, biocompatible NPs functionalized for drug-binding and pH-responsive delivery	[225,229,237,238]
Vaccine Adjuvants	*Halobacterium* sp., *Halorubrum tebenquichense*, *Deinococcus radiodurans*	GVNPs, archaeosomes, AuNPs	Antigen presentation, immune response stimulation, mucosal delivery potential	[237,239,242]
Biosensors	*Halococcus salifodinae*, *Halobacillus* sp., *Alkalibacillus* sp.	TeNPs, CdS QDs, ZnO NPs, AuNPs, AgNPs	High fluorescence, plasmon resonance, stability in saline, high sensitivity	[237,244,245]
Antioxidant Uses	*Actinobacteria*, *Desertifilum* sp., *Acinetobacter* sp., *Bacillus paramycoides*	MgO NPs, ZnO NPs, AuNPs, SeNPs	ROS scavenging, antihemolytic effects, low cytotoxicity, macrophage targeting	[247,248,249,250]
Antibacterial Uses	*Geobacillus* spp., *Deinococcus radiodurans*, *Haloferax* spp.	AgNPs, AuNPs, SeNPs	Membrane disruption, ROS generation, anti-biofilm, antibacteria	[232,252,253]

## Data Availability

Not applicable.
